# Retrospectively Estimating Energy Intake and Misreporting From a Qualitative Food Frequency Questionnaire: An Example Using Australian Cohort and National Survey Data

**DOI:** 10.3389/fnut.2021.624305

**Published:** 2021-04-07

**Authors:** James P. Goode, Kylie J. Smith, Michelle Kilpatrick, Monique Breslin, Wendy H. Oddy, Terence Dwyer, Alison J. Venn, Costan G. Magnussen

**Affiliations:** ^1^Menzies Institute for Medical Research, University of Tasmania, Hobart, TAS, Australia; ^2^Oxford Martin School and Nuffield Department of Obstetrics and Gynaecology, George Institute for Global Health, University of Oxford, Oxford, United Kingdom; ^3^Murdoch Children's Research Institute, Melbourne, VIC, Australia; ^4^Faculty of Medicine, Dentistry and Health Sciences, University of Melbourne, Melbourne, VIC, Australia; ^5^Research Centre of Applied and Preventive Cardiovascular Medicine, University of Turku, Turku, Finland; ^6^Centre for Population Health Research, Turku University Hospital, University of Turku, Turku, Finland

**Keywords:** portion size estimation, qualitative food frequency questionnaire, food composition database, energy intake, dietary misreporting, national survey data

## Abstract

Qualitative food frequency questionnaires (Q-FFQ) omit portion size information from dietary assessment. This restricts researchers to consumption frequency data, limiting investigations of dietary composition (i.e., energy-adjusted intakes) and misreporting. To support such researchers, we provide an instructive example of Q-FFQ energy intake estimation that derives typical portion size information from a reference survey population and evaluates misreporting. A sample of 1,919 Childhood Determinants of Adult Health Study (CDAH) participants aged 26–36 years completed a 127-item Q-FFQ. We assumed sex-specific portion sizes for Q-FFQ items using 24-h dietary recall data from the 2011–2012 Australian National Nutrition and Physical Activity Survey (NNPAS) and compiled energy density values primarily using the Australian Food Composition Database. Total energy intake estimation was daily equivalent frequency × portion size (g) × energy density (kJ/g) for each Q-FFQ item, summed. We benchmarked energy intake estimates against a weighted sample of age-matched NNPAS respondents (*n* = 1,383). Median (interquartile range) energy intake was 9,400 (7,580–11,969) kJ/day in CDAH and 9,055 (6,916–11,825) kJ/day in weighted NNPAS. Median energy intake to basal metabolic rate ratios were 1.43 (1.15–1.78) in CDAH and 1.35 (1.03–1.74) in weighted NNPAS, indicating notable underreporting in both samples, with increased levels of underreporting among the overweight and obese. Using the Goldberg and predicted total energy expenditure methods for classifying misreporting, 65 and 41% of CDAH participants had acceptable/plausible energy intake estimates, respectively. Excluding suspected CDAH misreporters improved the plausibility of energy intake estimates, concordant with expected body weight associations. This process can assist researchers wanting an estimate of energy intake from a Q-FFQ and to evaluate misreporting, broadening the scope of diet–disease investigations that depend on consumption frequency data.

## Introduction

Dietary assessment using food frequency questionnaires (FFQ) is common in large-scale epidemiological studies, largely due to practical necessity (1, 2). A semiquantitative FFQ collects consumption frequency and portion size information, facilitating subsequent estimates of nutrient intake when combined with a food composition database (1). Portion size information is usually embedded within frequency questions (3), or respondents have the additional option of answering “small” or “large” relative to a “medium” portion (4), or variations thereof (5). In contrast, a qualitative (Q)-FFQ omits any reference to portion size, thus restricting data collection to consumption frequency independent of quantity. While most studies use a semiquantitative FFQ, a 2002 review found that 22% of FFQs were qualitative (6). Investigators may adopt a Q-FFQ if deemed sufficient for their research objectives or to reduce respondent burden and simplify data processing (7–9); however, to estimate nutrient intake, researchers must specify a suitable portion size for each itemised food after the fact (2). The allocation of researcher-specified portion sizes to existing consumption frequency data appears acceptable for ranking individual food/nutrient intake (10–12), with consumption frequency shown to explain the majority of between-person variation in dietary intake (13, 14). Additionally, the distinction between “standard” (e.g., as recommended in the Australian Dietary Guidelines) and “typical” (i.e., actual consumption) portion sizes is important (15, 16), and greater clarity is needed in terms of operational definitions and the method of portion size computation.

A reasonable estimate of energy intake and dietary misreporting is integral to the analysis and interpretation of diet–disease relationships in epidemiological studies (17–21). Further, researchers should document how they handled the issue of energy adjustment and misreporting, as per reporting recommendations (22). Nutritional epidemiologists are principally interested in dietary composition (i.e., food and nutrient intake relative to total energy intake) (19, 21). Energy adjustment can help control for confounding, simulate an isocaloric dietary experiment, and reduce extraneous variation in food and nutrient intake (due to between-person differences in energy intake caused by non-dietary factors: body weight/composition, physical activity, and metabolic efficiency) (19–21). A secondary benefit is the “cancellation” of correlated measurement error between the nutrient of interest and energy intake, a consequence of deriving estimates from the same foods; thus, energy-adjusted nutrient intakes tend to be more valid, especially when errors are highly correlated (21, 23, 24). Dietary misreporting refers to inaccuracies arising from dietary assessment, where respondents under- or overreport true intake, and is routinely evaluated by contrasting subsequent estimates of energy intake against physiological expectations (18, 25, 26). The term energy misreporting (used hereafter) refers to improbable or physiologically implausible estimates of energy intake from dietary self-report. There is a widespread tendency towards the underestimation of energy intake in epidemiological studies (27). As such, failure to consider energy misreporting in diet–disease analyses may produce null or misleading associations. For example, the association between eating frequency and adiposity changes from null or inverse to positive following adjustment for energy misreporting (28, 29). Taken together, the absence of energy intake data may limit the investigation of diet–disease relationships that depend solely on frequency data collected using Q-FFQs.

To our knowledge, the literature lacks a comprehensive resource for researchers wanting to estimate energy intake and energy misreporting from a Q-FFQ. While possible (7), the estimation process is complicated by the need to specify suitable portion size information for the study population and each itemised food. Therefore, to support nutrition researchers in the use of Q-FFQ data to investigate diet–disease relationships in epidemiological studies, we provide an instructive example of Q-FFQ energy intake estimation that derives portion size information from a reference survey population and evaluates energy misreporting.

## Materials and Methods

**Figure 1:**
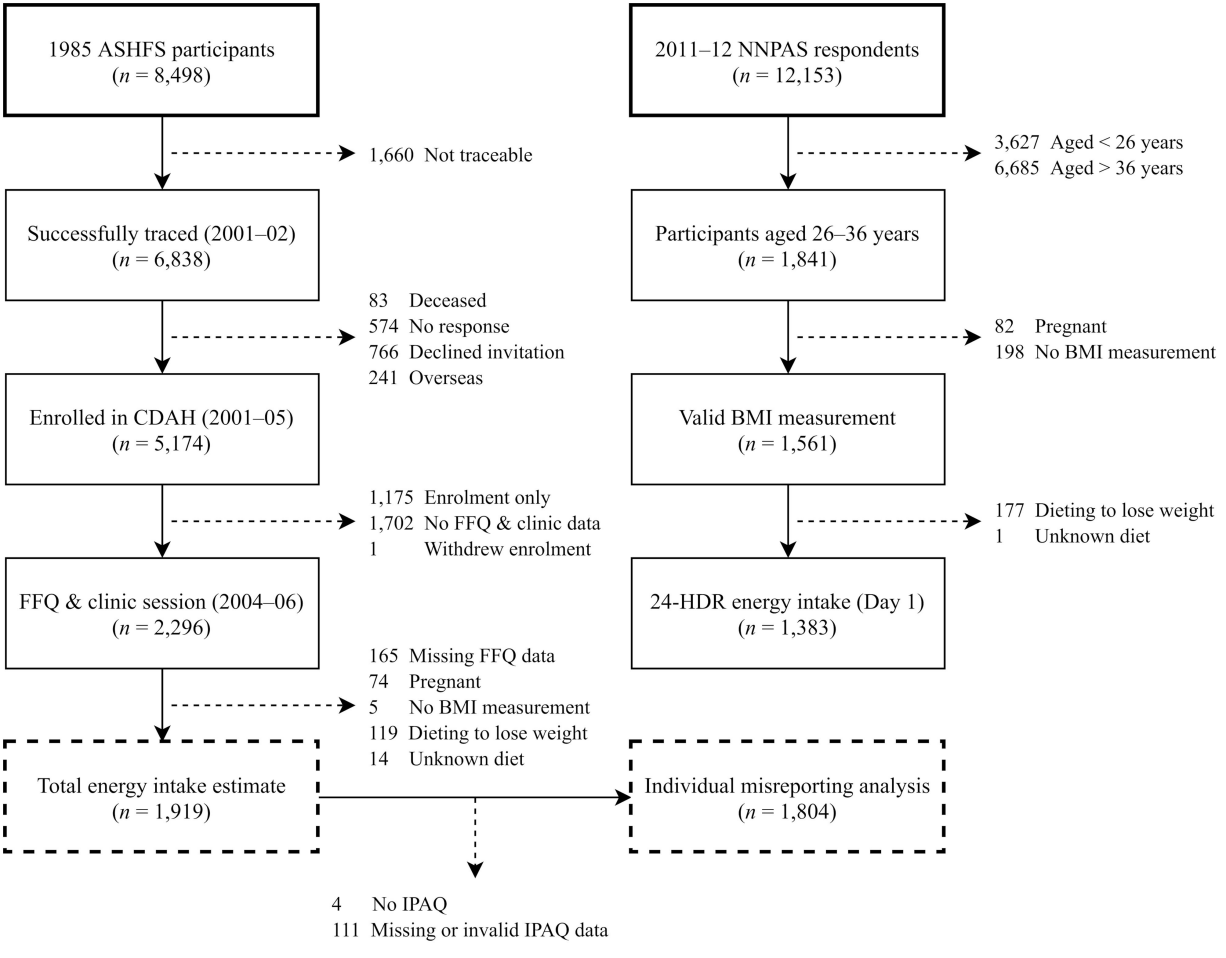
Participant flow chart for energy intake and misreporting analyses in CDAH and NNPAS. 24-HDR, 24-h dietary recall; ASHFS, Australian Schools Health and Fitness Survey; BMI, body mass index; CDAH, Childhood Determinants of Adult Health Study; FFQ, food frequency questionnaire; IPAQ, International Physical Activity Questionnaire.

The study population was a longitudinal cohort located in Australia named the Childhood Determinants of Adult Health Study (CDAH) (30). We estimated total energy intake and energy misreporting cross-sectionally at the 2004–2006 time point using Q-FFQ data. The 2011–2012 Australian National Nutrition and Physical Activity Survey (NNPAS) (31, 32) provided reference portion size information for Q-FFQ items and a benchmark for CDAH energy intake estimates. The Australian Bureau of Statistics provided NNPAS data as anonymized unit record files following access approval. Table 1 compares characteristics between CDAH and NNPAS participants of similar age (26–36 years), and Figure 1 documents participant selection into the study and subsequent estimates of energy intake and energy misreporting.

**Table 1:** Characteristics of CDAH and weighted NNPAS sample aged 26–36 years.

	**2004–2006 CDAH (*n* = 1,919)**			**2011–2012 NNPAS (*n* = 1,383)^a^**		
	**Men**	**Women**	***n***	**Men**	**Women**	***n***
*n* (%)	953 (50)	966 (50)	1,919	696 (50)	687 (50)	1,383
Age (year)	31.6 ± 2.6	31.4 ± 2.6	1,919	30.7 ± 3.2	30.9 ± 3.2	1,383
Body weight (kg)	85.7 ± 15.1	68.0 ± 15.1	1,919	83.9 ± 15.9	68.5 ± 16.0	1,383
Body mass index (kg/m^2^)	26.5 ± 4.3	24.7 ± 5.2	1,919	26.7 ± 4.4	25.4 ± 5.8	1,383
Normal, <25	372 (39)	630 (65)		247 (35)	396 (58)	
Overweight, 25–29.9	428 (45)	210 (22)		340 (49)	165 (24)	
Obese, ≥30	153 (16)	126 (13)		109 (16)	127 (18)	
Waist circumference (cm)	89.4 ± 10.7	77.7 ± 11.4	1,917	93.4 ± 11.7	83.2 ± 13.4	1,360
Total physical activity (min/week)^b^	200 (70–390)	200 (80–360)	1,804	180 (40–330)	160 (50–300)	1,363
AHS physical activity category^c^			1,804			1,363
High	206 (23)	165 (18)		162 (24)	83 (12)	
Moderate	266 (30)	311 (34)		193 (28)	229 (34)	
Low	281 (32)	359 (39)		211 (31)	263 (39)	
Sedentary	124 (14)	92 (10)		119 (17)	104 (15)	
Married (or living as married)	654 (69)	669 (69)	1,919	375 (54)	400 (58)	1,383
Highest education			1,914			1,369
University	369 (39)	475 (49)		262 (38)	297 (43)	
Vocational	341 (36)	232 (24)		271 (40)	229 (34)	
School	239 (25)	258 (27)		153 (22)	157 (23)	
Occupation			1,891			1,383
Professional/managers	554 (59)	497 (52)		260 (37)	221 (32)	
Non-manual	70 (7)	256 (27)		107 (15)	222 (32)	
Manual	285 (30)	43 (5)		265 (38)	60 (9)	
Unemployed/not in labor force	28 (3)	158 (17)		64 (9)	184 (27)	
Smoking status			1,915			1,383
Never	540 (57)	545 (57)		362 (52)	394 (57)	
Former	174 (18)	221 (23)		168 (24)	153 (22)	
Current	238 (25)	197 (21)		166 (24)	140 (20)	
Self-reported health rating			1,907			1,383
Excellent	150 (16)	152 (16)		145 (21)	129 (19)	
Very good	380 (40)	418 (44)		280 (40)	274 (40)	
Good	335 (35)	324 (34)		214 (31)	218 (32)	
Fair	72 (8)	59 (6)		45 (6)	51 (7)	
Poor	11 (1)	6 (1)		12 (2)	14 (2)	

*Data are mean ± standard deviation, median (interquartile range) or n (%)*.

*AHS, Australian Health Survey; CDAH, Childhood Determinants of Adult Health Study; NNPAS, National Nutrition and Physical Activity Survey*.

a *Characteristics weighted to N = 2,669,458 using Australian Bureau of Statistics survey weights. Thus, characteristics are Australian population estimates derived from a sample of n = 1,383 NNPAS respondents (696 men and 687 women)*.

b *Total physical activity undertaken for fitness, recreation or sport, or walking for transport in last week*.

c *Physical activity categories historically used in Australian Health Survey outputs, providing a comparative descriptor of overall physical activity and intensity between CDAH and weighted NNPAS: activity duration (min) × intensity factor (walking for fitness or transport = 3.5, moderate intensity = 5, vigorous intensity = 7.5), with the following cutoffs: sedentary (<50, includes no physical activity), low (50–<800), moderate (800–1,600 or >1,600 and <1 h vigorous physical activity), and high (>1,600 and ≥1 h vigorous physical activity)*.

### CDAH

The 1985 Australian Schools Health and Fitness Survey (ASHFS) collected fitness, well-being, and performance data from a nationally representative sample of Australian children aged 7–15 years (*n* = 8,498). Full sampling and data collection procedures are available elsewhere (33, 34). Researchers traced survey participants in 2001–2002 using electoral rolls, the Australian National Death Index, telephone directories, and school and family networks (35). In 2001–2005, researchers invited successfully traced individuals (*n* = 6,838) to form the CDAH study, aimed at determining childhood predictors of cardiovascular disease and diabetes later in life. In total, 5,174 traced participants enrolled in CDAH. In 2004–2006, participants (aged 26–36 years) attended one of 34 clinics around Australia and returned a mailed Q-FFQ (*n* = 2,296), thus forming the first 20-year follow-up of ASHFS participants. Each State's Director-General of Education approved the completion of ASHFS. The Southern Tasmania Health and Medical Human Research Ethics Committee approved CDAH and the first 2004–2006 follow-up of ASHFS participants (H6020), with all participants providing written informed consent.

### Food Frequency Questionnaire

In total, 2,885 CDAH participants self-administered a 127-item Q-FFQ, a response rate of 55.8% for enrolled participants. Participants completed and retuned mailed Q-FFQs periodically across all seasons between 2004 and 2006. Supplementary Table 1 lists Q-FFQ items. The Q-FFQ used in CDAH is a modified version of the 107-item Q-FFQ used in the 1995 National Nutrition Survey (36), which was originally based on the 121-item Q-FFQ developed for the baseline phase of the Melbourne Collaborative Cohort Study (7) using the Harvard FFQ model (3). CDAH's Q-FFQ assesses average long-term patterns of dietary intake, as per its predecessors, with changes from the National Nutrition Survey FFQ largely including the addition of new items (e.g., seafood, nuts and seeds, and alcoholic drinks). Participants reported their average frequency of consumption of each Q-FFQ item over the previous 12 months. The Q-FFQ did not specify or collect portion size information for items. The nine possible response options ranged from “never or less than once a month” to “6+ times per day” (Supplementary Table 2). Participants reported certain mixed foods as distinct items (e.g., *salad*, or *vegetable casserole*) while separating others into component foods (e.g., sandwich into *white bread, ham/bacon*, and *green/mixed salad*, etc.). For seasonal foods, the Q-FFQ asked for average consumption while in season. Participants also completed a food habits questionnaire, which included questions on their usual way of eating; how often they used reduced-fat dairy products, spreads, and oily dressings; the removal of visible fat from meat; whether sugar is added to tea and coffee; and milk type preference (e.g., whole, reduced fat, skimmed, soy, etc.).

### Anthropometry

Clinic technicians measured CDAH participants' body weight using portable scales (Heine, Dover, NH, USA), height using a stadiometer (Invicta, Leicester, UK), and waist circumference using a steel (non-stretch) anthropometric tape (Lufkin, Apex Tool Group, Sparks, MD, USA) at the narrowest point between the iliac crest and 10th rib. Body mass index (BMI) is body weight in kilogrammes divided by height in metres squared. We apply World Health Organisation BMI classifications (37). Due to few underweight CDAH participants (*n* = 29), we combined underweight and normal-weight BMI categories.

### Physical Activity

CDAH participants self-administered the validated, long-form International Physical Activity Questionnaire (IPAQ) (38). IPAQ assesses the frequency, duration, and intensity of four physical activity domains over the preceding week: occupation, transport, household, and leisure. When evaluating energy misreporting, participants were assigned a low, moderate, or high physical activity category according to IPAQ data processing and scoring protocols (39). In addition, to allow a direct comparison of physical activity between CDAH and NNPAS participants, physical activity categories historically used in Australian Health Surveys outputs (31) were calculated from IPAQ data (Table 1).

### Data Management

Initially, 2,296 CDAH participants self-administered the mailed Q-FFQ and attended a clinic session. Participants were excluded if they had ≥20 blank Q-FFQ items (40), an entire page of blank items, or were missing food habit questionnaire responses (*n* = 165), were pregnant (*n* = 74), had no BMI measurement (*n* = 5), or were following a “weight reduction diet” (*n* = 119) or an unknown dietary practise (*n* = 14). We employed zero imputation for Q-FFQ items left blank, assuming no consumption (40). Blank Q-FFQ items accounted for 0.4% of total responses across participants. Overall, 1,919 CDAH participants had their total energy intake estimated, but four did not return IPAQ and 111 had missing or invalid IPAQ data, preventing the assignment of a physical activity category (Figure 1). Therefore, assessment of individual-level energy misreporting was restricted to 1,804 CDAH participants.

We first removed the Q-FFQ item *water* since it does not contribute to energy intake, resulting in 126 items. Q-FFQ consumption frequencies were converted to daily equivalent frequencies using Supplementary Table 2. The consumption frequency “never or less than once per month” was coded as zero, as it was considered more reflective of no consumption. We applied a seasonal fruit adjustment to three Q-FFQ items to account for periods of no, minimal, or reduced availability using publicly available supply calendars from two Australian supermarkets (Supplementary Table 3).

### NNPAS

NNPAS (32), one of the three national health surveys conducted as part of the 2011–2013 Australian Health Survey (31), was completed between May 2011 and June 2012. The aim was to establish national benchmark information on nutrition to allow food and nutrient adequacy assessments and historical comparisons. A stratified multistage sampling of urban and rural private dwellings obtained a representative sample of Australians (*n* = 12,153). In total, 9,519 private dwellings participated (77% household response rate). Random sampling identified one adult aged ≥18 years from each dwelling and, if applicable, one child aged 2–17 years. Trained interviewers performed data collection during a face-to-face household visit using a computer-assisted approach. The age range of respondents was 2–85+ years, with 188 respondents over the age of 84 classified as 85 years. NNPAS is the largest and most comprehensive nutrition survey performed to date in Australia. Full details of the complex survey design are available elsewhere (31). The Census and Statistics Act, 1905 provided the Australian Bureau of Statistics with the authority to conduct NNPAS, with all respondents providing written informed consent.

### Twenty-Four-Hour Dietary Recall

NNPAS respondents completed up to two 24-h dietary recalls (24-HDR) with a trained interviewer using a modified version of the automated multiple-pass method adapted to the Australian food system (31). The Agricultural Research Service developed the automated multiple-pass method (41), which is a computerised format designed to maximise dietary recall and reduce respondent burden, with >10,000 preprogrammed foods. Participants completed a face-to-face 24-HDR interview (day 1, *n* = 12,153), followed by a second 24-HDR at least 8 days later by telephone (day 2, *n* = 7,735), reporting all food and drink consumed from midnight to midnight the day before. Typical portion sizes were estimated visually using the United States Department of Agriculture's Food Model Booklet (31, 42), with images replaced by Australian-sourced food and beverage containers. Interviewers conducted 24-HDRs across all seasons and days of the week to capture seasonal and day-to-day variation in eating patterns.

A code-based classification system, specifically developed for the 2011–2013 Australian Health Survey, catalogued and organised all reported foods (31, 43). Unique (or analogous) foods were assigned an individual (eight-digit) survey ID and then sorted into hierarchical groups based on similarity and designated a grouping code: minor (four–five-digit), submajor (three-digit), and major (two-digit). An example classification would be the food *orange, navel* (survey ID, 16301001) and its associated hierarchical groups: *oranges* (minor, 16301), *citrus fruit* (submajor, 163), and *fruit products and dishes* (major, 16). Individual food items also possess a food combination code documenting culinary use (e.g., milk added to cereal).

### Anthropometry

Trained interviewers measured NNPAS respondents' body weight using digital scales, height using a stadiometer, and waist circumference using a metal (non-stretch) measuring tape at the midpoint between the iliac crest and last palpable rib in the midaxillary line (31). The Australian Health Survey 2011–2013 users' guide does not specify further equipment details. Due to few underweight NNPAS respondents (*n* = 32), we combined underweight and normal-weight BMI categories.

### Physical Activity

NNPAS respondents answered interviewer questions on physical activity and exercise undertaken over the preceding week (31). Questions focused on several activity domains: walking for fitness or recreation/sport, walking for transport, and moderate and vigorous intensity physical activity and exercise (excluding walking and household chores). Respondents recalled the number of sessions and total time spent engaging in each physical activity and exercise session. Aspects of the NNPAS physical activity questionnaire are based on the Active Australia Survey (44), but respective formats are not directly comparable.

### Data Management

Initially, 1,841 NNPAS respondents aged 26–36 years had a 24-HDR (day 1) total energy intake estimate. Subsequent exclusions included pregnancy (*n* = 82), no BMI measurement (*n* = 198), and dieting to lose weight (*n* = 177) or following an unknown dietary practise (*n* = 1). Overall, total energy intake was estimated for 1,383 NNPAS respondents (Figure 1).

### Energy Intake

A single 24-HDR can be used to describe mean intake at the population level but often results in a wider distribution (1) shifted to the left (45). The use of mean energy intake from both 24-HDRs (days 1 and 2) would have resulted in a 36% smaller sample size (*n* = 888). Since we are interested in population-level intake, and not usual intake at the individual level (1), we estimated NNPAS energy intake only using day 1 24-HDR data. For reference, mean 24-HDR energy intake from day 2 (8,847 kJ/day) is 7.5% lower than that from day 1 (9,563 kJ/day).

### Portion Size Database

Each CDAH Q-FFQ item was matched to entries from the 2011–2013 Australian Health Survey food classification system (31, 43). Matching was frequently performed at the individual survey ID and minor food group level by combining similar entries in terms of variety and preparation method. We first disaggregated nested items (e.g., *orange or mandarin or grapefruit*) into subcomponents and specified subcomponents for non-specific items (e.g., *other fruit not listed*) using personal judgement. The Q-FFQ does not specify whether vegetables had been cooked; therefore, for common vegetable items that are often eaten raw or cooked (*spinach, mushrooms, carrots, capsicum, tomatoes*, and *onion or leek*), we included raw and cooked components. The Q-FFQ includes 126 items (72 single and 54 multiple-component items) that were systematised into 238 components.

We estimated sex-specific portion sizes for Q-FFQ components using dietary data from NNPAS respondents aged 19–69 years who completed both 24-HDRs (days 1 and 2). We defined a typical portion size as the usual amount of food in grammes consumed on a single eating occasion. When participants consumed the same food on more than one occasion, the arithmetic mean of all eating occasions was used. The use of both 24-HDRs helps improve the representation of each respondent's diet concerning “usual” food intake. To achieve an adequate sample of NNPAS respondents, we combined portion size data for men and women for uncommon Q-FFQ components with low NNPAS response rates (e.g., *coconut, mussels or oysters*, and *liver*).

When deriving portion size estimates from NNPAS 24-HDR data, we used medians for single component Q-FFQ items and weighted arithmetic means (of medians) for multiple-component items. Medians were the measure of central tendency as portion size data usually exhibited right-skew distributions. Weighting for multiple-component items was sex specific and accounted for relative component frequency (i.e., common foods contributed more than uncommon foods when estimating portion sizes). Overall, portion size information was sex specific for 99 Q-FFQ items (79%), non-sex specific for 10 items (8%), and a combination thereof for 17 multiple-component items (13%). Supplementary Figure 1 documents the selection of NNPAS respondents for portion size estimates. We provide examples of the matching and portion size estimation process for CDAH women in Table 2, regarding Q-FFQ items, the Australian Health Survey food classification system, and NNPAS-derived portion size estimates. Our customised portion size database is available as a Microsoft Excel file (Supplementary Material).

**Table 2:** Examples of FFQ item portion size and energy density creation for CDAH women.

**2004–2006 CDAH**	**2011–2013 AHS food classifications**		**2011–2012 NNPAS**				**Density^a^**
**FFQ item *Component***	**Code**	**Description**	***N***	**Portion (g)**	***n***	**Weight (%)**	**kJ/100 g**
Banana	16501001	Banana, cavendish, peeled, raw	2,152	98	876	n/a	394
Orange/grapefruit/mandarin				102^b^			184^b^
*Orange*	16301001	Orange, navel (all varieties), peeled, raw	2,152	131	224	41	175
*Grapefruit*	16303002	Grapefruit, peeled, raw	4,090	258	23	2	125
*Mandarin*	16303003	Mandarin, peeled, raw	2,152	75	304	56	195
Other fruit				63^b^			395^b^
*Cherry*	16403003	Cherry, raw	2,152	82	34	10	250
*Fig*	16503003	Fig, fresh, peeled or unpeeled, raw	4,090	75	25	4	195
*Passionfruit*	16504007	Passionfruit, raw	4,090	18	25	4	304
*Kiwi*	16601008	Kiwifruit, gold, peeled or unpeeled, raw	2,152	78	110	34	237
*Avocado*	24705001	Avocado, raw	2,152	51	157	48	579
Carrots				35^b^			148^b^
*Raw*	24301005	Carrot, mature, fresh or frozen, raw	2,152	26	251	34	141
*Cooked*	24301006	Carrot, mature, fresh or frozen, baked, roasted	2,152	39	488	66	152
Mixed red meat dishes				281^b^			608^b^
*Casserole (beef)*	18701001	Casserole, commercial, beef, and vegetable	2,152	275	111	36	493
*Curry (beef)*	18701013	Curry, commercial, beef, tomato sauce	2,152	330	46	15	597
*Stir-fry (beef)*	18701027	Stir-fry, commercial, beef	2,152	258	59	19	688
*Mixed lamb dishes*	18705	Lamb dishes w/ gravy, sauce or vegetables	2,152	358	62	20	687
*Mixed pork dishes*	18708	Pork dishes w/gravy, sauce or vegetables	2,152	125	31	10	697
Milk added to coffee/tea^c^							
Full fat	19101	Milk, cow, fluid, regular whole, full-fat	2,152	31	892	n/a	281
Reduced fat	19103	Milk, cow, fluid, reduced fat, <2 g/100 g	2,152	31	747	n/a	191
Nonfat	19105	Milk, cow, fluid, skim, nonfat	2,152	31	447	n/a	146
Oil and vinegar dressing^c^							
Full fat	23303	Italian and French-style dressings, full fat	2,152	22	297	n/a	1,206
Reduced or non-fat	23304	Italian and French-style dressings, reduced/non-fat	2,152	22	73	n/a	108
Lamb (roast, chop)^c^							
Untrimmed fat	18102	Lamb and mutton	2,152	104	255	n/a	1,053
Trimmed fat	18102	Lamb and mutton	2,152	104	255	n/a	804

*N denotes the total number of NNPAS women (n = 2,152), or men and women (n = 4,090), aged 19–69 years who provided dietary intake data. n denotes the number of respondents who reported consuming each FFQ item/component. It was often necessary to combine multiple AHS food classifications and AFCD/AUSNUT entries, but for illustrative purposes, we present one code/description per FFQ item/component. Portion sizes are medians, and energy densities (kJ/100 g) are unweighted means, unless stated otherwise. Weight (%) indicates the relative contribution of each component to weighted means for multiple-component FFQ items*.

*AFCD, Release 1 Australian Food Composition Database; AHS, Australian Health Survey; AUSNUT, 2011–2013 Australian Food and Nutrient Database; CDAH, Childhood Determinants of Adult Health Study; FFQ, food frequency questionnaire; NNPAS, National Nutrition and Physical Activity Survey*.

a *Energy density values were primarily derived from AFCD and supplemented with AUSNUT when necessary or for mixed dishes*.

b *Weighted mean based on relative component frequency (i.e., common foods contribute more than uncommon foods). Portion sizes use sex-specific weightings, while energy density values use the average weight between men and women*.

c *Food habit questionnaire responses informed suitable energy density value allocation when estimating total energy intake (e.g., how regularly participants used reduced-fat dairy products, spreads, and oily dressings, removed visible fat from meat, and their milk type preference)*.

### Energy Density Database

Each CDAH Q-FFQ component was matched to Release 1 (January 2019) Australian Food Composition Database entries, an update of NUTTAB (nutrient tables) 2010 (46). We supplemented this process with the 2011–2013 Australian Food and Nutrient Database (AUSNUT) when a suitable match was not possible or the Q-FFQ component was a mixed dish (e.g., *vegetable casserole*) (43). We matched Q-FFQ mixed dishes to predefined AUSNUT recipes. The Australian Food Composition Database and AUSNUT express energy and nutrient values per 100 g edible portion for food and beverages. It was frequently necessary to combine similar database entries in term of variety and preparation method during the matching process. A discussion of the differences between the Australian Food Composition Database (formerly NUTTAB) and AUSNUT is available elsewhere (47). For consistency, we primarily constructed our energy density database using the Australian Food Composition Database, which principally includes laboratory analysed foods, whereas data sources vary in AUSNUT.

Food habit questionnaire responses allowed for the modification of 15 Q-FFQ items. For each item, energy density values accounted for whether, and how often, a participant used reduced-fat products, removed visible fat from meat, added sugar to tea and coffee, and the type of milk they usually consumed. When generating composite energy density values (kJ/g), we calculated arithmetic means for single-component Q-FFQ items and weighted arithmetic means for multiple-component items. Weighting accounted for relative component frequency and used the average weighting of men and women, as used in portion size estimates. Thus, energy density values apply to both men and women. We provide examples of the energy density value generation process for Q-FFQ items in Table 2 using our customised energy density database, which is available as a Microsoft Excel file (Supplementary Material).

### CDAH Energy Intake

CDAH total energy intake (kJ/day) estimation was as follows: daily equivalent frequency × portion size (g) × energy density value (kJ/g) for each Q-FFQ item, summed. As our energy density database expressed energy content per 100 g, it was necessary to first divide energy density values by 100 before estimating total energy intake. Energy intake estimates include energy provided by dietary fibre fermentation. Box 1 summarises our approach for estimating energy intake from Q-FFQ data by externally deriving portion size information from NNPAS.

Box 1 **Estimating qualitative FFQ energy intake using NNPAS-derived portion size information.**

Step 1: Match each FFQ item to entries from the 2011 to 2013 Australian Health Survey food classification system

- Disaggregate nested items (e.g., *orange or mandarin or grapefruit*) into subcomponents
- Specify subcomponents for non-specific items using personal judgment (e.g., *other fruit not listed*)
- Create raw and cooked subcomponents where culinary appropriate (e.g., *carrots, tomatoes*, and *onion or leek*)

*Note:* Matching primarily accomplished at the individual (8-digit) ID level (*FOODCODC*) by combining multiple entries, or by using/combining minor (*FIVDIGC*) and submajor (*THRDIGC*) food groups

step 2: Derive portion size estimates from 2011 to 2012 NNPAS using 24-HDR portion size data (*GRAMWGT*)

- Include respondents aged 19–69 years (*AGEC*) who completed both 24-HDRs (*DAYNUM*)
- Exclude eating occasions (*EATOCC*) classified as “Extended consumption” or “Not determined”
- Use the mean of all eating occasions for respondents who consumed the same food on more than one occasion
- Assign a food combination code (*COMBCODE*) to specific use items/components (e.g., *milk added to breakfast cereal*), apply ABS day 2 person weights (*NPAD2WGT*), and then generate sex-specific (*SEX*) median portion sizes for each FFQ item/component
- Combine male and female portion size data for uncommon FFQ items/components (e.g., *coconut* and *liver*)
- Calculate weighted means for multiple-component FFQ items (e.g., *apple or pear*) according to relative component frequency (i.e., common components contribute more than uncommon components)

Step 3: Match each FFQ item/component to Release 1 Australian Food Composition Database entries

- When necessary, supplement with 2011–2013 AUSNUT database entries (e.g., unmatched items, mixed dishes)

*Note*: Matching primarily accomplished by combining similar entries in terms of variety and preparation method

Step 4: Generate energy density values (kJ/g)

- Generate mean energy density values for single component FFQ items
- Calculate weighted means for multiple-component FFQ items according to relative component frequency
- Modify FFQ items to account for food habit questionnaire responses (e.g., reduced-fat food usage, removal of visible fat from meat, milk type preference, and sugar added to tea/coffee). For example, the item *milk as a drink* had unique energy density values for whole milk, skimmed milk, soy milk, etc.

Step 5:CDAH data management

- Exclude participants with missing FFQ data (≥20 items, ≥1 blank page, missing food habit questionnaire responses), who are pregnant, have no body mass index measurement, or are following a diet for weight loss purposes
- Employ zero imputation for FFQ items left blank, which assumes no consumption
- Convert FFQ responses to daily equivalent frequencies (e.g., “2–3 times per day” recoded to 2.5)
- Apply a seasonal fruit adjustment to select items to account for periods of no, minimal, or reduced availability

Step 6: CDAH total energy intake estimate

- Estimate energy intake (kJ/day) as follows: daily equivalent frequency × portion size (g) × energy density value (kJ/g) for each FFQ item, summed
- Use age-matched (*AGEC*) 2011–12 NNPAS energy intake estimates (*ENERGYT1*) with ABS day 1 person weights (*NPAFINWT*) as a benchmark for CDAH estimates

Note: ABS microdata variable identifiers (ITALICS) are provided for user convenience.

24-HDR, 24-h dietary recall; ABS, Australian Bureau of Statistics; AUSNUT, Australian Food and Nutrient Database; CDAH, Childhood Determinants of Adult Health Study; FFQ, food frequency questionnaire; NNPAS, National Nutrition and Physical Activity Survey.

### Energy Misreporting

Energy misreporting, where energy intake estimates appear to conflict with physiological expectations, was investigated in CDAH using the Goldberg (25, 48) and predicted total energy expenditure (pTEE) methods (26, 49). Each method applies principles of energy physiology, predictive equations, and confidence limits (accounting for measurement error and variance) to evaluate energy misreporting at the group and individual levels. Individuals below and above estimated confidence limits are suspected under- and overreporters, respectively, with remaining individuals considered acceptable/plausible reporters. In NNPAS, we only investigated group-level energy misreporting using the Goldberg method. A single 24-HDR estimate of energy intake creates a wide distribution, so when assessing misreporting at the individual level, it limits the ability to detect true under- and overreporters (25). In addition, NNPAS did not assess occupation-related physical activity, which hinders the allocation of a suitable physical activity level to respondents. Therefore, we did not conduct an individual-level assessment of energy misreporting in NNPAS. A detailed discussion of the Goldberg and pTEE method is available elsewhere (18), with our full calculations provided as Supplementary Material.

### Goldberg Method

The Goldberg method applies the estimated energy intake to basal metabolic rate (EI:BMR) ratio. The Mifflin–St. Jeor equation estimated basal metabolic rate using sex, body weight, height, and age (50). In large samples (*n* > 500), researchers assess group-level misreporting by directly comparing the EI:BMR ratio with the expected (or known) physical activity level of the study population. The physical activity level is an expression of energy requirement, often defined as total energy expenditure divided by basal metabolic rate over a 24-h period. The Goldberg method fundamentally assumes that energy intake approximates total energy expenditure during periods of body weight stability. The population (median) physical activity level of 1.63 identified by the Scientific Advisory Committee on Nutrition (p. 21) served as a reference point when evaluating group-level misreporting in CDAH and NNPAS, which is suggestive of a low activity lifestyle (51). Assessment of individual-level misreporting compares the EI:BMR ratio to a confidence limit about a specified physical activity level (while accounting for measurement error and variance in estimated energy intake and basal metabolic rate, and energy requirement). The calculated confidence limit (usually ±2 SD) identifies energy intake estimates at the extremes of the distribution (i.e., statistically unlikely).

### pTEE Method

The pTEE method applies the estimated energy intake to total energy expenditure (EI:TEE) ratio. Total energy expenditure is estimated using prediction equations published by the Institute of Medicine (52) that utilise sex, BMI categories, physical activity, body weight, and height. The assessment of group-level misreporting is performed by comparing the EI:TEE ratio to a value of 1.0, which represents energy balance (i.e., body weight stability), with values <1.0 signifying underreporting and >1.0 overreporting. Assessment of individual-level misreporting compares the EI:TEE ratio to a percentage-based confidence limit (estimated using known measurement error and variance in estimated energy intake and total energy expenditure, and energy requirements). The pTEE method advocates a more stringent statistical cutoff (±1–1.5 SD) than the Goldberg method, intending to create a more biologically plausible sample where energy intake estimates are consistent with principles of energy physiology (18, 53).

### Energy Intake Plausibility

**Figure 2:**
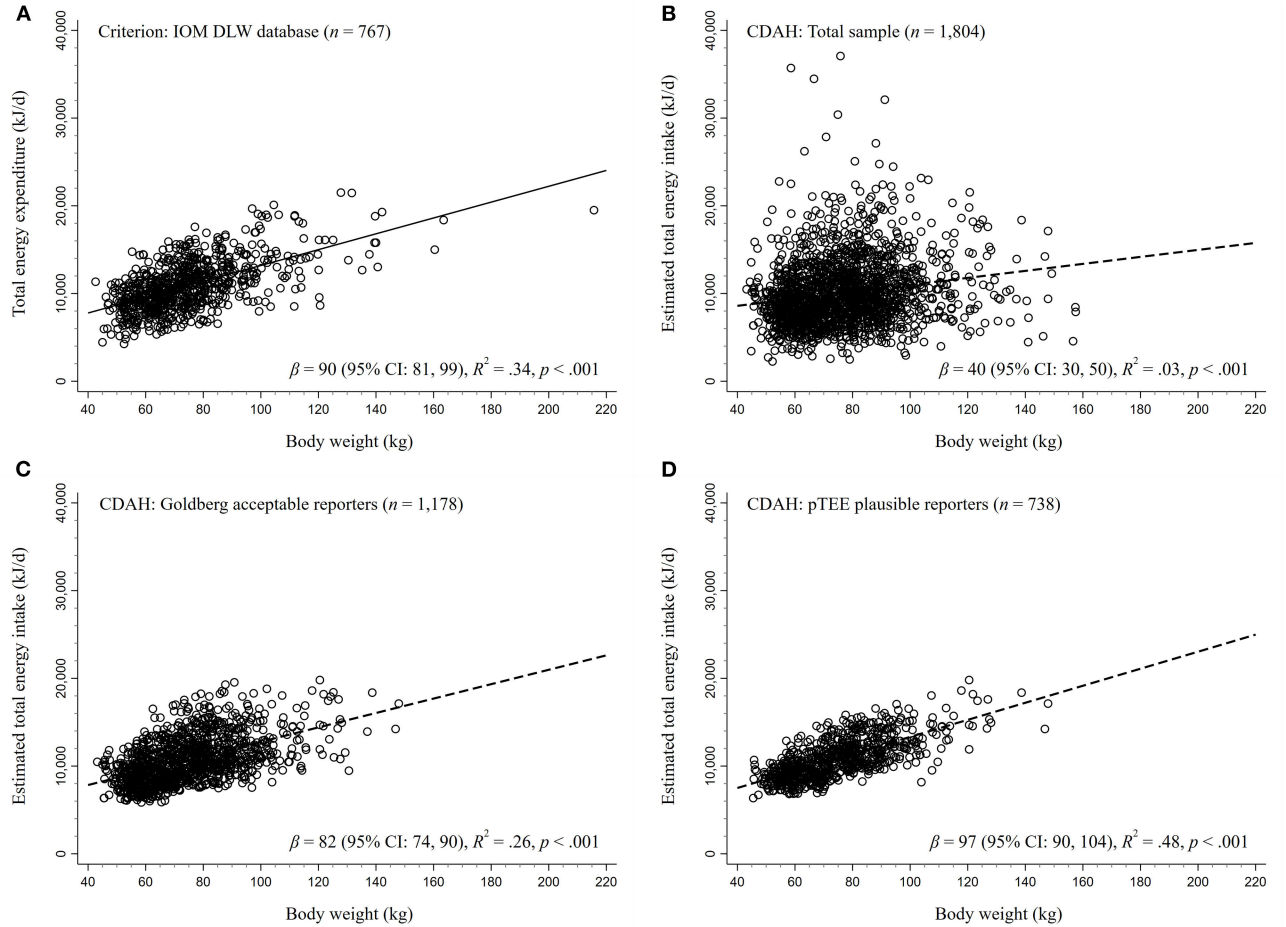
Regressions of energy expenditure and estimated energy intake on body weight. The DLW database compiled for the 2002 Dietary Reference Intakes consensus report published by IOM served as the criterion for **(A)** the association between measured total energy expenditure and body weight. Regressions of estimated energy intake on body weight in CDAH were performed on **(B)** the total sample (no exclusions), **(C)** acceptable Goldberg method energy reporters (±2 standard deviation cutoff, misreporters excluded), and **(D)** pTEE method plausible energy reporters (±1.5 standard deviation cutoff, misreporters excluded). In theory, since energy intake should equal (or approximate) energy expenditure in weight stable individuals, beta-coefficients (β) and coefficients of determination (*R*^2^) similar to the criterion imply that estimates are physiologically plausible. CDAH, Childhood Determinants of Adult Health Study; CI, confidence interval; DLW, doubly labeled water; IOM, Institute of Medicine; pTEE, predicted total energy expenditure method.

The Goldberg and pTEE methods aim to create a more physiologically plausible sample by identifying energy intake estimates consistent with principles of energy physiology. In theory, total energy intake should equal (or approximate) total energy expenditure during periods of weight and body composition stability. There is an established association between measured total energy expenditure and body weight, with total energy expenditure tending to increase alongside body weight (54). Therefore, in a physiologically plausible dataset, the association between estimated energy intake and measured total energy expenditure on body weight should be approximately equal, as used in the pTEE method (26). We assessed the plausibility of CDAH energy intake estimates by comparing the regression model for energy intake on body weight with a reference criterion model that regressed measured total energy expenditure on body weight. The metrics of comparison include the beta-coefficient (β), coefficient of determination (*R*^2^), and level of significance. The doubly labelled water database compiled by the Institute of Medicine for establishing Dietary Reference Intakes (52) provided a criterion regression for measured total energy expenditure on body weight (Figure 2A). The database consists of 767 free-living adults aged ≥19 years who are predominantly from the US and Netherlands (44% men) with a BMI ≥18.5 kg/m^2^ and excludes pregnant and lactating women (52). A description of the doubly labelled water technique for measuring total energy expenditure is available elsewhere (55).

### Data Analysis

The matching of Q-FFQ items/components to Australian Health Survey food classification codes and food composition database entries, and typical portion size and energy density value generation, was performed in Microsoft Excel. Total energy intake estimates, assessments of energy misreporting, and regression analyses were conducted in Stata 16.2 (StataCorp 2019, Texas, USA). Composite portion size and energy density calculations use arithmetic means.

### NNPAS Person Weights

NNPAS-derived estimates include person weights provided by the Australian Bureau of Statistics (i.e., the number of population units represented by each respondent). NNPAS benchmarked weights against the 2006 Census of Population and Housing using the following calibration levels: sex, age group, area of residence, and season (31). Thus, NNPAS respondents provide Australian population estimates. We applied initial person weights (*NPAFINWT*) to 24-HDR energy intake (day 1) and characteristic estimates, accounting for all respondents who completed the first face-to-face interview. Conversely, we applied day 2 person weights (*NPAD2WGT*) to NNPAS-derived portion size estimates, since we analysed data from respondents who completed both 24-HDR interviews (day 1, face to face; day 2, telephone).

## Results

### Participant Characteristics

CDAH and weighted NNPAS characteristics are broadly comparable (Table 1). Notably, CDAH participants had slightly less overweight and obesity, lower waist circumferences, a higher prevalence of marriage (including living as married) and professional/managerial employment, and reported engaging in modestly higher levels of physical activity.

### Energy Intake

In Table 3, median energy intake estimates were 3.7% higher in CDAH (9,400 kJ/day) than weighted NNPAS (9,055 kJ/day) and, separately, 9.3% higher for men and 6.5% higher for women. Overweight and obese participants in CDAH and weighted NNPAS had consistently similar or lower energy intake estimates than normal-weight participants.

**Table 3:** Estimated energy intake in CDAH and weighted NNPAS sample aged 26–36 years.

**Cohort**	**Sex/*BMI class***	***n***	**Mean ± SD**	**Median (IQR)**	**Range**	**EI:BMR^a^**
2004–2006 CDAH	Men	953	11,758 ± 4,214	11,105 (8,899–13,931)	2,485–37,074	1.46 (1.17–1.83)
	*Normal*	372	11,935 ± 4,416	11,464 (9,121–13,707)	4,557–37,074	1.57 (1.26–1.91)
	*Overweight*	428	11,466 ± 3,904	10,756 (8,782–13,703)	2,485–32,093	1.38 (1.12–1.77)
	*Obese*	153	12,144 ± 4,510	11,286 (9,044–14,696)	2,944–27,182	1.33 (1.08–1.67)
	Women	966	8,604 ± 2,856	8,193 (6,682–9,922)	2,256–35,702	1.41 (1.14–1.74)
	*Normal*	630	8,692 ± 3,017	8,205 (6,725–10,007)	2,256–35,702	1.49 (1.22–1.86)
	*Overweight*	210	8,470 ± 2,538	8,213 (6,566–9,713)	3,176–17,149	1.37 (1.07–1.59)
	*Obese*	126	8,382 ± 2,507	8,061 (6,623–9,811)	4,294–17,412	1.18 (0.92–1.40)
	**Overall**	**1,919**	**10,170** ± **3,925**	**9,400 (7,580**–**11,969)**	**2,256**–**37,074**	**1.43 (1.15**–**1.78)**
2011–12 NNPAS^b^	Men	696	10,733 ± 4,088	10,122 (7944–12,828)	1230–30,316	1.36 (1.05–1.72)
	*Normal*	246	10,739 ± 4,134	10,168 (7,873–12,795)	1,230–26,447	1.50 (1.13–1.86)
	*Overweight*	346	10,706 ± 3,782	10,068 (8,007–12,691)	2,332–25,747	1.32 (1.04–1.70)
	*Obese*	104	10,802 ± 4,866	10,538 (7,919–13,268)	1,518–30,316	1.25 (0.93–1.60)
	Women	687	8,114 ± 3,093	7,678 (5,848–9,794)	972–24,592	1.33 (1.00–1.76)
	*Normal*	380	8,366 ± 3,116	8,116 (6,030–10,026)	972–23,088	1.49 (1.15–1.90)
	*Overweight*	169	7,939 ± 3,067	7,453 (5,723–9,620)	1,400–16,012	1.19 (0.99–1.56)
	*Obese*	138	7,556 ± 2,984	7,115 (5,319–9,400)	2,278–24,592	1.04 (0.79–1.33)
	**Overall**	**1,383**	**9,563** ± **3,899**	**9,055 (6,916**–**11,825)**	**972**–**30,316**	**1.35 (1.03**–**1.74)**

*BMI classifications (kg/m^2^): normal, <24.9; overweight, 25–29.9; and obese, ≥30. Basal metabolic rate estimated from the Mifflin–St. Jeor equation using sex, body weight, height, and age. Total energy intake is in kJ/day (divide by 4.184 for kcal)*.

*BMI, body mass index; CDAH, Childhood Determinants of Adult Health Study; EI:BMR, estimated energy intake to basal metabolic rate; IQR, interquartile range; NNPAS, National Nutrition and Physical Activity Survey; SD, standard deviation*.

a *Values are median (IQR)*.

b *Characteristics weighted to N = 2,669,458 using Australian Bureau of Statistics survey weights. Thus, total energy intakes are Australian population estimates derived from a sample of n = 1,383 NNPAS respondents aged 26–36 years (696 men and 687 women)*.

### Group-Level Misreporting

Overall, weighted NNPAS reported a lower median EI:BMR ratio than CDAH (1.35 vs. 1.43), with normal-weight participants reporting higher EI:BMR ratios (1.49–1.57) than obese participants (1.04–1.33) in both samples (Table 3). Men consistently reported higher EI:BMR ratios than women, with the biggest difference occurring among obese individuals. Based on measured total energy expenditure, a median population physical activity level (EI:BMR) is typically 1.63, which is generally reflective of a low activity lifestyle, with lower values associated with increasingly more sedentary behaviour (51). In terms of a lower limit reference point, immobile individuals with a sustainable, weight stable lifestyle may have an EI:BMR ratio as low as 1.2 (56). In total, 29.2% of CDAH participants and 38.4% of NNPAS respondents had an EI:BMR below 1.2. However, to account for the wider distribution resulting from a single 24-HDR energy intake estimate, an EI:BMR below 0.9 is arguably more suitable as a lower limit in NNPAS (25) and instead corresponds to 16.0% of respondents.

### Individual-Level Misreporting

In Table 4, the Goldberg method identified 65% (*n* = 1,178) of CDAH participants as acceptable reporters, while the pTEE method identified 41% (*n* = 738) as plausible reporters. Across both energy misreporting methods, normal-weight participants reported the highest percentage of acceptable/plausible reporters, while underreporting was most prevalent in overweight and obese participants. Overreporting was most common in normal-weight participants, particularly for the pTEE method (17–19%). The median EI:TEE ratio in CDAH was 0.85 and, separately, 0.86 for men and 0.84 for women, with obese participants reporting the lowest EI:TEE ratios. Therefore, according to the pTEE method, we underestimated median energy intake in CDAH by 15%, since an EI:TEE ratio of 1.0 implies energy balance (i.e., body weight stability).

**Table 4:** Percentage of energy misreporters in CDAH using the Goldberg and pTEE methods.

		**Goldberg method (±2 SD)**				**pTEE method (±1.5 SD)**			
**Sex/BMI class**	***n***	**U_**R**_ (%)**	**A_**R**_ (%)**	**O_**R**_ (%)**	**EI:BMR^a^**	**U_**R**_ (%)**	**P_**R**_ (%)**	**O_**R**_ (%)**	**EI:TEE^a^**
Men	877	27.9	65.3	6.7	1.44 (1.15–1.81)	42.3	42.1	15.2	0.86 (0.69–1.07)
*Normal*	350	22.0	68.3	9.7	1.56 (1.26–1.90)	32.9	47.7	19.4	0.92 (0.74–1.14)
*Overweight*	388	29.9	65.7	4.4	1.38 (1.12–1.71)	46.4	40.7	12.9	0.83 (0.68–1.03)
*Obese*	139	37.4	56.8	5.8	1.29 (1.00–1.66)	57.6	31.7	10.8	0.77 (0.61–0.99)
Women	927	29.2	65.3	5.5	1.41 (1.14–1.74)	46.1	39.8	14.1	0.84 (0.69–1.05)
*Normal*	600	23.0	70.0	7.0	1.48 (1.21–1.85)	39.3	43.5	17.2	0.89 (0.73–1.11)
*Overweight*	203	32.5	64.5	3.0	1.37 (1.07–1.61)	50.3	37.9	11.8	0.81 (0.67–0.98)
*Obese*	124	54.0	43.6	2.4	1.18 (0.92–1.40)	71.8	25.0	3.2	0.71 (0.57–0.90)
**Overall**	**1,804**	**28.6**	**65.3**	**6.1**	**1.43 (1.15**–**1.77)**	**44.5**	**40.9**	**14.6**	**0.85 (0.69**–**1.06)**

*Goldberg and pTEE method classifications: UR, underreporter; OR, overreporter; AR, acceptable reporter; and PR, plausible reporter. BMI classifications (kg/m^2^): normal, <24.9; overweight, 25–29.9; and obese, >30. The total percentage of energy misreporting may not equal 100% due to rounding. The pTEE method ±1.5 SD cutoff corresponds to an estimated energy intake at ±19% of pTEE*.

*BMI, body mass index; CDAH, Childhood Determinants of Adult Health Study; EI:BMR, energy intake to estimated basal metabolic rate; EI:TEE, energy intake to predicted total energy expenditure; pTEE, predicted total energy expenditure; SD, standard deviation*.

a *Values are median (interquartile range)*.

### Energy Intake Plausibility

In Figures 2A–D, we present a series of estimated energy intake on body weight regressions in CDAH, alongside our reference criterion from an independent sample: measured total energy expenditure on body weight (β = 90 kJ/kg, *R*^2^ = 0.34; *p* < 0.001). Thus, β is change in estimated energy intake or measured total energy expenditure (kJ/day) per 1 kg increase in body weight. A β and *R*^2^ approximating the criterion (Figure 2A) would lend support to the physiological plausibility of CDAH energy intake estimates, in line with theoretical expectations. In the total CDAH sample, there was a relationship between estimated energy intake and body weight (β = 40 kJ/kg, *R*^2^ = 0.03, *p* < 0.001), with body weight explaining little variance in energy intake (Figure 2B), but this relationship did not approximate the criterion. Figure 2C demonstrates how the exclusion of energy misreporters identified using the Goldberg method (*n* = 626) results in an estimated energy intake on body weight relationship that approximates the criterion (*β* = 82 kJ/kg, *R*^2^ = 0.26, *p* < 0.001). Similarly, the exclusion of energy misreporters identified using the pTEE method (*n* = 1,066) also produced a relationship that approximated, although modestly exceeded, the criterion (*β* = 97 kJ/kg, *R*^2^ = 0.48, *p* < 0.001), with body weight explaining almost half of the variance in energy intake (Figure 2D).

### Effect Modification

Due to effect modification, we stratified regressions of measured total energy expenditure and estimated energy intake on body weight by sex (Supplementary Figures 2, 3). Stratifying by sex eliminated the association between estimated energy intake and body weight in the total sample of CDAH men (*β* = 3 kJ/kg, *R*^2^ = 0.00, *p* = 0.72) and women (*β* = −9 kJ/kg, *R*^2^ = 0.00, *p* = 0.14) and moderately attenuated the *β* and *R*^2^ in subsequent regressions that excluded suspected CDAH energy misreporters. However, as before, regressions more closely resembled the criterion following the exclusion of energy misreporters identified using either the Goldberg or pTEE method, and particularly the pTEE method.

## Discussion

We provide an instructive example of Q-FFQ energy intake estimation followed by an assessment of energy misreporting, including the creation of an accompanying portion size and energy density database. While the majority of studies use a semiquantitative FFQ to assess dietary intake in epidemiological studies (6), researchers may decide to use a Q-FFQ for simplicity or to reduce respondent burden (7–9). As such, our paper is a helpful resource for researchers wanting to use frequency data to investigate diet–disease relationships, namely, generation of suitable portion size information and the practise of constructing a Q-FFQ-linked portion size and energy density database. Applying our approach, we obtained a reasonable but crude estimate of Q-FFQ energy intake in a nationally representative sample of Australians (CDAH) and quantified the extent of energy misreporting. The benchmarking of CDAH energy intake estimates against Australian population estimates from a national nutrition survey (NNPAS) provided a simple indicator of face validity. As expected, we found notable energy underestimation in CDAH and weighted NNPAS, particularly among the overweight and obese. The exclusion of suspected CDAH energy misreporters improved the plausibility of energy intake estimates, in line with physiological expectations.

The decision of whether or not to collect portion size information during dietary assessment has proved a contentious issue (2, 6). Examples of commonly used semiquantitative FFQs, or variations thereof, include the Harvard FFQ (3), where a reference portion size is specified within frequency questions, and the Block-National Cancer Institute FFQ (4), which additionally allows respondents to indicate “small” or “large” relative to a “medium” portion size. Therefore, to estimate energy intake from Q-FFQ data, researchers must make portion size assumptions once data collection is complete. Compared to a reference instrument, correlation coefficients for energy intake appear equivalent between a Q-FFQ and a semiquantitative FFQ that specifies reference portion sizes (0.44 vs. 0.42), as reviewed by Cade et al. (12). For instance, Pietinen et al. developed and validated a 44-item Q-FFQ against food records (12 × 2 days) for use in a large interventional trial of Finnish men (11). The intention was to monitor dietary changes over time and rank participants according to their relative nutrient intake. Following two Q-FFQ administrations 6 months apart, researchers assumed “average” portions sizes *post hoc* (except for bread). While the Q-FFQ underestimated energy intake by 3,314 kJ/day compared to food records, correlation coefficients were low to moderate (*r* = 0.43–0.45), and overall ranking agreement was reasonable (lowest or adjacent quintile, 57–71%). The application of portion size information to existing consumption frequency data appears a viable research option and, in terms of ranking energy intake, demonstrates comparable performance to a semiquantitative FFQ that specifies a reference portion size alongside frequency questions.

The use of uniform FFQ portion sizes (i.e., a one-size-fits-all approach) appears acceptable for ranking participants according to energy and nutrient intake since frequency of consumption explains most between-person variance in dietary intake (13, 14). A large German study (*n* = 26,764) by Noethlings et al. investigated the impact of assuming “medium” portion sizes for a 148-item semiquantitative FFQ on between-person variance in food group intake, as opposed to using incremental portion size information (e.g., small, medium, or large) (13). Medium-portion sizes were based on fixed household measures or medians derived from the German National Nutrition Survey. Despite differences in absolute intake (g/day) for most food groups across sex, age, and BMI categories, frequency of consumption explained the majority of intake variance (mean *R*^2^ = 84%; range, 71–93%). Overall, loss of variance due to assuming medium-portion sizes ranged from 7 to 29%, with further stratification by sex, age, and BMI offering no appreciable improvement in explanatory power (*R*^2^ < 1%). The observation that frequency of consumption is the primary contributor to absolute intake is supported by an early work of Heady (14) who developed a “short-cut method” for classifying dietary intake in large prospective studies during the 1960s.

The collection of additional portion size information from FFQ respondents (e.g., whether small, medium, or large), above simply specifying a single reference portion size, is generally considered advantageous (6). Molag et al. investigated the issue of semiquantitative FFQ design in a 2007 meta-analysis of 40 studies (57). Overall, pooled correlation coefficients (*r*) for energy intake against a reference instrument (usually 24-HDR or food records) was 0.45 (range, 0.16–0.77). The authors reported modestly higher correlation coefficients when researchers sought additional portion size information from respondents, as opposed to specifying or assuming a “standard” portion (0.52 vs. 0.46). Therefore, obtaining additional portion size information may provide a small improvement in participant ranking according to energy intake. While the use of a single reference or assumed portion size will inevitability reduce within-person variation, FFQ development usually focuses on food and nutrient ranking ability (i.e., capturing between-person variation) (3–5). Researchers must balance the decision to obtain additional portion size information with increased respondent burden and whether the advantages outweigh the disadvantages (2).

In the absence of validation studies, the validity of our Q-FFQ is unclear. The Q-FFQ used in CDAH is a second-generation version of the Melbourne Q-FFQ (7) that was administered during the baseline phase (1990–1994) of the Melbourne Collaborative Cohort Study (58). That said, Ireland et al. have established the relative validity of the Melbourne Q-FFQ, which was developed using Weighed Food Survey data (7). The Weighed Food Survey involved a volunteer sample of 810 men and women aged 40–69 years living in Melbourne, Australia who completed weighed food records (2 × 4 days). In Australian-born Melbourne Collaborative Cohort Study participants, Melbourne Q-FFQ median energy intake was 8,320 kJ/day in men (*n* = 6,522) and 7,100 kJ/day in women (*n* = 4,202), corresponding to a 21 and 4% underestimation compared to Weighed Food Survey volunteers. Estimates excluded participants with improbable energy intakes and energy provided by alcoholic drinks and added sugar. Overall, Melbourne Collaborative Cohort Study participants had a median EI:BMR ratio of 1.25, indicating marked underreporting. In comparison, we reported a median EI:BMR ratio of 1.43 in CDAH, but after excluding improbable energy intakes (*n* = 91) and items relating to alcoholic drinks and added sugar from estimates, the EI:BMR became 1.29. While a direct comparison is complicated by differences in study design, date of completion, analytical approach, and population characteristics, each Q-FFQ version (CDAH and Melbourne) appears to underestimate group-level energy intake to a similar degree.

Our approach underestimated median energy intake in CDAH by 15% when compared to predicted total energy expenditure (10% within-person error). In a pooled analysis of five validation studies, Freedman et al. found semiquantitative FFQs to underestimate energy intake by 31% in men and 28% in women (range, 24–32%) relative to doubly labelled water measured total energy expenditure (45). More recently, a systematic review by Burrows et al. found semiquantitative FFQs to underestimate energy intake anywhere from 5 to 42% (*n* = 19), with the majority of studies reporting an underestimation of 20–30% (59). Thus, our underestimation of energy intake in CDAH is consistent with previous findings; however, the extent of underreporting is arguably less severe than expected. Freedman et al. also observed a consistent association between BMI (30 vs. 25 kg/m^2^) and increased underreporting severity (45), concordant with our findings in CDAH. The exclusion of suspected CDAH energy misreporters, identified using either the Goldberg or pTEE method, improved the plausibility of our energy intake estimates, in agreement with pTEE method proof-of-concept findings (26). We adopted an intermediate cutoff (±1.5 SD) when applying the pTEE method, which identified 59% of participants as energy misreporters. While a more stringent cutoff may be advantageous (e.g., ±1 SD), researchers must balance any improvement in the plausibility of dietary data with sample size loss (26) and the possible introduction of selection bias, especially when a predictor of underreporting is also associated with the outcome (e.g., diet–obesity relationships).

The benchmarking of CDAH energy intake estimates against Australian population estimates derived from NNPAS respondents provides a useful indicator of face validity (60). NNPAS interviewers administered up to two 24-HDRs to survey respondents using the automated multiple-pass method, a computerised format designed to maximise dietary recall and reduce respondent burden. Energy intake estimates obtained using the automated multiple-pass method (mean of three 24-HDRs) has been validated against doubly labelled water measured total energy expenditure (61). In weight-stable, free-living adults aged 30–70 years (*n* = 524), the automated multiple-pass method underestimated mean energy intake by 10% in men and 12% in women and to a greater extent among overweight and obese participants. The mean estimated energy intake to measured resting energy expenditure ratio (similar but distinct from EI:BMR) was 1.43. We found a comparable degree of energy underreporting in weighted NNPAS (EI:BMR = 1.35) and the same pattern of underreporting across BMI classifications. However, such a comparison assumes similarity in study design, analytical approach, and population characteristics. In NNPAS, the automated multiple-pass method was performed within expectations in terms of energy intake estimation, but our underestimation appears more severe. The use of three 24-HDRs and greater portion size estimation assistance in the automated multiple-pass method validation study may partly explain the lower degree of energy underreporting to what we found in NNPAS. A further consideration is the reported increase in energy underreporting between the 1995 Australian National Nutrition Survey and 2011–2013 NNPAS (31, 62). A rise in obesity may partly explain this change, but the underlying reason remains unclear. In NNPAS, a high BMI category is the strongest and most consistent predictor of energy underreporting (62)—a near-ubiquitous finding in national dietary surveys (63). While other factors are also associated with energy underreporting (e.g., eating restraint and social desirability), as reviewed previously (27, 63–66), a more thorough examination of the characteristics of underreporters in CDAH and NNPAS was beyond the scope of our paper.

Our study and overall approach to estimating Q-FFQ energy intake in our study population (CDAH) has important limitations. We fundamentally assume that each Q-FFQ eating occasion involved a typical portion size, and NNPAS-derived portion sizes were representative of CDAH participants. Although, the concept of a “typical” portion size is somewhat tenuous because within-person variance often exceeds between-person variance for most foods (16, 67). The process of creating a portion size and energy density database for Q-FFQ items was labour intensive and often involved arbitrary decisions, such as deciding whether and how to organise items into subcomponents and which Australian Health Survey classification codes and food composition database entries to match to Q-FFQ items/components. The original purpose of CDAH's Q-FFQ was to assess average patterns of long-term dietary intake and not to accurately estimate absolute food/nutrient intake. Retrospectively applying portion size information to existing consumption frequency data does not change how participants interpreted and answered the original questionnaire. That is, CDAH participants reported average consumption frequencies without a reference portion size. Furthermore, since our Q-FFQ is a second-generation implementation of the Melbourne Q-FFQ developed in the early 1990s (7), its current content validly may have decreased due to changes in Australian consumption patterns over time. It was not always possible to generate sex-specific portion sizes for uncommon Q-FFQ items due to low NNPAS response rates, partially reducing representativeness; however, these 10 items only account for 0.7% of estimated total energy intake in CDAH. While sex contributes little to overall variance in FFQ determined food intake, absolute intake (g/day) does vary by sex, with men generally reporting higher intakes than women (13). The Q-FFQ did not collect information on item variety, making it difficult to generate an appropriate energy density value for certain items. For instance, some participants may consume basa (426 kJ/100 g), while others consume salmon (1,212 kJ/100 g), yet the item *fresh fish* had a composite energy density of 1,011 kJ/100 g. However, in the absence of such information, we mitigated the impact of multiple-component Q-FFQ items with highly variable energy content among components by weighting according to relative component frequency. National consumption patterns may have changed during the interval between Q-FFQ administration (2004–2006) and NNPAS completion (2011–2012), potentially reducing the representativeness of NNPAS-derived portion size estimates. In Australia, the typical portion size of discretionary foods, with an appreciable contribution to energy intake, has changed between 1995 and 2011–2012 (68). As such, we may have under- or overestimated the proportion of energy from “discretionary foods,” which may account for up to 34% of total energy intake in CDAH (data not shown). While a single 24-HDR can be tentatively used to assess mean population intake (1), direct comparison with a FFQ warrants caution since FFQ-derived intakes are prone to greater systematic error, usually towards underestimation (45, 69). Finally, participants self-reported physical activity using IPAQ, which has fair-to-moderate criterion validity (38), increasing the risk of misclassifying energy misreporters because the Goldberg and pTEE methods do not account for physical activity level assignment error (18). While an objective measure of physical activity is preferable (e.g., wearable monitors), such methods are not always feasible in large-scale cohort studies due to implementation costs and participant and researcher burden (70).

Notwithstanding limitations, our study and overall approach has notable strengths. Our portion size database accounts for sex differences in absolute food intake (13, 16) and reflects Australian consumption patterns by using national survey data (NNPAS)—as recommended (71) and pioneered by Block et al. (4). In support of generalizability, researchers often benefit from the availability of portion size data from national surveys and their accompanying food composition databases, for example, the USA National Health and Nutrition Examination Survey (72) and the UK National Diet and Nutrition Survey (73). In addition, numerous European countries possess nationally representative survey data on nutrition (74). We benchmarked CDAH energy intake estimates against Australian population estimates derived from NNPAS to help judge face validity, alongside a meticulous assessment of energy misreporting using two established methods: Goldberg and pTEE. We evaluated the plausibility of CDAH energy intake estimates using the known association between measured total energy expenditure and body weight as a criterion, as per the pTEE method (26). Our approach used *typical* portion size information, reflecting actual intake as opposed to recommended “standard” serves (15, 16). For example, the 2013 Australian Dietary Guidelines generically defines one serving of fresh fruit as 150 g (75). In our portion size database, the median portion size across fruit items was 104 g with a range of 58–276 g. NNPAS respondents estimated portion sizes with visual support from the United States Department of Agriculture's Food Model Booklet, assisting accuracy (42). That said, the ability of respondents to accurately recall portion size information (i.e., measurement error) has posed a longstanding challenge in dietary research (76). The incorporation of food habit questionnaire responses (e.g., reduced-fat food usage and milk type preference) aided in the appropriate allocation of energy density values when estimating CDAH energy intake. Finally, IPAQ assessed all physical activity domains and assigned low, moderate, or high physical activity categories to CDAH participants (39), supporting the allocation of suitable physical activity level values when assessing energy misreporting, as recommended by Black (25). The sensitivity of the Goldberg method for detecting individual-level underreporting improves when unique physical activity level values (e.g., 1.49, 1.63, and 1.78) are assigned across physical activity categories (e.g., low, moderate, and high), in contrast to using a single physical activity level of 1.55 across all participants (77).

In energy balance and obesity research, many consider the use of energy intake derived from dietary self-report inappropriate due to excessive measurement error (78). The Energy Balance Measurement Working Group reaffirmed this position in 2015 (79). Weight change, on the other hand, is suggested as a more practical and suitable alternative to energy intake for assessing energy balance in free-living populations (80). However, the working group also appeared sceptical of the demonstrated value of self-reported dietary data (79), prompting a letter to the editor (81) and an author response (82). In the background of such uncertainty, prominent researchers have published articles to reaffirm the value of nutritional epidemiology, address common misunderstandings (83, 84), and propose good practise recommendations (17) while also recognising the challenge of dietary measurement error. When investigating diet–disease relationships, and as a general principle, it is recommended that models include some form of energy adjustment to help improve risk estimation—even if using error-prone estimates of energy intake (17, 85). Different approaches to energy adjustment and their application have been discussed in detail elsewhere (19–21), along with procedures for identifying energy misreporters (18). After determining the prevalence and extent of energy misreporting, researchers must carefully consider how to account for suspected misreporters in analyses (e.g., exclusion, stratification, or adjustment). For guidance, several studies have compared different approaches for handling energy misreporters in analyses of diet- and obesity-related outcomes (86–88). George Beaton and colleagues encapsulate issue of self-reported dietary data well: “There will always be error in dietary assessments. The challenge is to understand, estimate, and make use of the error structure during analysis” (89). Thus, when thoughtfully applied and interpreted, sensible estimates of energy intake can play a fundamental role in the investigation of diet–disease relationships in epidemiological studies.

## Conclusions

Through an instructive example, we provide a resource for researchers using frequency data to investigate diet–disease relationships. We outline the process undertaken to estimate energy intake and misreporting from a Q-FFQ. This required the linking of food items to a custom-made portion size and energy density database, which we include as Supplementary Material to support transparency. To inform database creation, an analysis of national survey data (NNPAS) provided suitable portion size information, and two Australian food composition databases supplied energy density values—data sources readily available to most Australian-based researchers, although our approach is generalizable since researchers often have access to national nutrition survey and food composition data from their respective countries. In our example, we obtained a reasonable, albeit crude, estimate of Q-FFQ energy intake in a representative sample of Australian adults and quantified the extent of energy misreporting. As anticipated, notable group-level underreporting was present in CDAH and weighted NNPAS, especially among overweight and obese individuals. The exclusion of suspected CDAH energy misreporters created a more physiological plausible sample; consequently, if considered necessary, one must consider the trade-off between improving the plausibility of dietary data and sample size loss. Our approach is unsuitable if researchers require an accurate measure of energy intake, given the inherent challenge and inevitable measurement error of assessing self-reported diet in large populations of free-living individuals. However, in terms of ranking ability, estimates may suffice for investigations of dietary composition (i.e., energy-adjusted intakes) and for mitigating the influence of energy misreporting. When applying portion size information to frequency data, researchers should clearly define operational definitions and describe the method of portion size computation, including the origin of underlying data. In addition, greater transparency regarding the creation of portion size and food composition databases linked to FFQs would be beneficial. Considering the importance of energy adjustment and misreporting in nutritional epidemiology, our paper may help broaden the scope of diet–disease investigations that currently depend on frequency of consumption data.

## Data Availability Statement

The original contributions presented in the study are included in the article/Supplementary Material, further inquiries can be directed to the corresponding author/s.

## Ethics Statement

Each State's Director-General of Education approved the 1985 Australian Schools Health and Fitness Survey. A parent or guardian provided informed consent for each participant. The Southern Tasmania Health and Medical Human Research Ethics Committee approved the Childhood Determinants of Adult Health Study and the first follow-up of survey participants (H6020). The Census and Statistics Act 1905 provided the Australian Bureau of Statistics with the authority to conduct the 2011-2012 National Nutrition and Physical Activity Survey and includes a provision for data release. Study and survey participation was contingent on obtaining written informed consent.

## Author Contributions

JG conceived the study design, performed data collection and analysis, and draft the manuscript. KS provided advice on the analysis of dietary data and database construction. MB provided statistical and coding assistance. CM supervised study completion. All authors critically reviewed draft manuscripts, suggested revisions, and approved publication.

## Conflict of Interest

The authors declare that the research was conducted in the absence of any commercial or financial relationships that could be construed as a potential conflict of interest.

## Abbreviations

ASHFS: Australian Schools Health and Fitness Survey
AUSNUT: Australian Food and Nutrient Database
BMI: body mass index
CDAH: Childhood Determinants of Adult Health Study
EI:BMR: estimated energy intake to basal metabolic rate
EI:TEE: estimated energy intake to total energy expenditure
FFQ: food frequency questionnaire
IPAQ: International Physical Activity Questionnaire
NNPAS: National Nutrition and Physical Activity Survey
pTEE: predicted total energy expenditure
Q-FFQ: qualitative food frequency questionnaire
SD: standard deviation
24-HDR: 24-h dietary recall.
