# Supplementary material for: Retrospectively Estimating Energy Intake and Misreporting From a Qualitative Food Frequency Questionnaire: An Example Using Australian Cohort and National Survey Data
Source: Front Nutr. 2021 Apr 7;8:624305. doi: 10.3389/fnut.2021.624305 (PMC8058357; doi:10.3389/fnut.2021.624305)
Supplement: Supplementary file 2 [file Data_Sheet_2.docx]

# Tables

Table S1. Food items included in CDAH’s qualitative food frequency questionnaire (*n* = 127)

| **Dairy foods** | **Dressing and spreads (cont.)** |
| --- | --- |
| Flavoured milk drink (e.g., milkshake, iced coffee, hot chocolate) | Vegemite™, Marmite™ or Promite™ |
| Milk as a drink | Creamy dips and spreads |
| Milk in hot beverages (e.g., in coffee, tea) | **Non-dairy** |
| Milk added to breakfast cereal | Fruit juice (100%) |
| Cream or sour cream | Vegetable, tomato juices |
| Ice-cream | Fruit juice drink or fruit cordial |
| Yoghurt, plain or flavoured (incl. fromage frais) | Low-joule cordial |
| Cottage or ricotta cheese | Cordial |
| Cream cheese (e.g., Philadelphia™) | Low-joule soft drink |
| Cheddar and other cheeses | Soft drinks (incl. flavoured mineral water) |
| **Bread & cereal foods** | Water (incl. unflavoured mineral water, soda water, tap water) |
| White bread, toast, or rolls | Coffee—full strength |
| Wholemeal/mixed grain bread, toast, or rolls | Decaffeinated coffee |
| English muffin, bagel, or crumpet | Tea—green or black (ordinary tea) |
| Flatbread (e.g., pita, chapatti) | Herbal tea |
| Dry or savoury biscuits, crispbread, crackers | Soy beverages |
| Muesli | Light beer |
| Cooked porridge | Medium strength beer |
| breakfast cereal | Full strength beer |
| Rice (white or brown) | Red wine |
| Pasta (incl. filled), noodles | White wine or champagne/sparkling wine |
| **Meat, fish, eggs** | Wine cooler |
| Mince dishes (e.g., rissoles, meatloaf) | Spirit-based mixed drinks (e.g., Lemon Ruski™) |
| Mixed dishes with beef, veal, lamb, pork (e.g., casserole, stir-fry) | Sherry/port/fortified wines |
| Beef, veal—roast, chop, or steak | Spirits, liquors |
| Lamb—roast, chop | Other alcoholic drinks (e.g., cider) |
| Pork—roast, chop | **Vegetables (incl. frozen and canned)** |
| Ham/bacon | Green/mixed salad (incl. lettuce, tomato etc.) in a sandwich |
| Luncheon meats, salami | Green/mixed salad (incl. lettuce, tomato etc.) as a side-salad with a main meal |
| Sausages (beef, pork, other) | Stir-fried or mixed vegetables |
| Liver (incl. pâté) | Vegetable casserole |
| Other offal (e.g., kidneys) | Potato—boiled, mashed, or baked |
| Mixed dishes with chicken, duck, turkey (e.g., casserole, stir-fry) | Hot chips/roast potatoes/potato wedges |
| Chicken, turkey, duck—roast, steamed or barbequed | Sweet potato |
| Canned fish (e.g., tuna, salmon, sardines) | Pumpkin |
| Fresh fish—steamed, baked, grilled | Peas (incl. snow peas) |
| Frozen fish—steamed, baked, grilled | Green beans |
| Fish, fried | Silverbeet/spinach |
| Mussels/oysters | Broccoli |
| Lobster/crayfish/yabbies | Cauliflower |
| Calamari/squid | Brussel sprouts, cabbage, coleslaw |
| Prawns | Carrots |
| Other seafood | Mushrooms |
| Egg | Capsicum |
| **Sweets, baked goods, and snacks** | Sweetcorn, corn on the cob |
| Cakes, sweet muffins, scones, or pikelets | Zucchini, eggplant, squash |
| Sweet pies or sweet pastries | Cucumber |
| Other pudding or desserts | Tomatoes (except when in ‘mixed salad’) |
| Plain, sweet biscuits | Lettuce (except when in a ‘mixed salad’) |
| Cream, chocolate biscuits | Celery (except when in ‘mixed salad’) |
| Meat pie, sausage roll or other savoury pastries | Onion or leek |
| Pizza | Soybeans, tofu |
| Hamburger | Baked beans |
| Chocolate (incl. chocolate bars e.g., Mars bar™) | Other beans, lentils |
| Other confectionary | **Fruits** |
| Potato chips, corn chips, Twisties™ etc. | Fruits—dried, frozen, canned |
| Almonds, walnuts, hazelnuts | Fresh fruit salad |
| Cashews | Apple or pear |
| Coconuts | Orange, mandarin, grapefruit |
| Peanuts | Banana |
| Pistachio | Peach, nectarine, plum or apricot |
| Seeds—pumpkin, sesame, pine nuts, tahini | Mango or paw-paw |
| Other nuts, seeds | Pineapple |
| **Dressing and spreads** | Grapes or berries |
| Oil and vinegar dressing | Melon (watermelon, rockmelon, or honeydew melon) |
| Mayonnaise or other creamy dressings | Other fruit not listed |
| Jam, marmalade, syrup, or honey |  |
| Peanut butter, other nut spreads |  |

*Note*. Items are grouped and listed in order of appearance in the food frequency questionnaire.

Table S2. Food frequency questionnaire response options to daily equivalent frequencies conversions

| FFQ response option | Daily equivalent frequency |
| --- | --- |
| Never or less than once a month | 0 |
| 1–3 times per month | 0.07 |
| Once per week | 0.14 |
| 2–4 times per week | 0.43 |
| 5–6 times per week | 0.78 |
| Once per day | 1 |
| 2–3 times per day | 2.5 |
| 4–5 times per day | 4.5 |
| 6+ times per day | 6 |

*Note.* Calculations used one calendar year (365 days), the mean number of days per week (7.02) and month (30.42), and the mid-point of ranged response options.

Table S3. Food frequency questionnaire seasonal fruit adjustment calculations

| FFQ item  *Components* | Months where fruit is readily available ^a^ | | |  | **Availability**  Weighting (%) |
| --- | --- | --- | --- | --- | --- |
|  | Coles ^b^ | Woolworths ^b^ | Average |  |  |
| Peach, nectarine, apricot or plum |  |  |  |  | **7.5 months** |
| *Peach or nectarine* | 9 | 7 | 8 |  | 69 |
| *Plum* | 6 | 8 | 7 |  | 24 |
| *Apricot* | 5 | 4 | 4.5 |  | 8 |
| Mango or papaya |  |  |  |  | **8.3 months** |
| *Mango* | 7 | 8 | 7.5 |  | 82 |
| *Papaya* | 12 | – | 12 |  | 18 |
| Other fruit not listed |  |  |  |  | **8.7 months** |
| *Cherry* | 5 | 4 | 4.5 |  | 10 |
| *Fig* | 9 | – | 9 |  | 6 |
| *Passionfruit* | 12 | – | 12 |  | 6 |
| *Kiwi* | 4 | 6 | 5 |  | 34 |
| *Avocado* | 12 | 12 | 12 |  | 45 |

*Note.* We applied a seasonal fruit adjustment to three FFQ items to account for periods of no, minimal or reduced availability using publicly available supply calendars from two Australian supermarket chains: Coles and Woolworths. Childhood Determinants of Adult Health Study participants reported usual in-season fruit intake over the preceding 12 months. We estimated the actual number of months where FFQ items are readily available. Weighting (%) accounts for relative component frequency since FFQ items were multiple-component (i.e., common components contribute more than uncommon components). We adjusted FFQ items as follows: (daily equivalent frequency ÷ 12) × weighted months of availability.

*Abbreviations:* FFQ, food frequency questionnaire.

^a^ Sourcing 100% Australian or a combination of Australian and imported.

^b^ Coles data current as of May 2019 and Woolworths data current as of June 2020.

# Figures


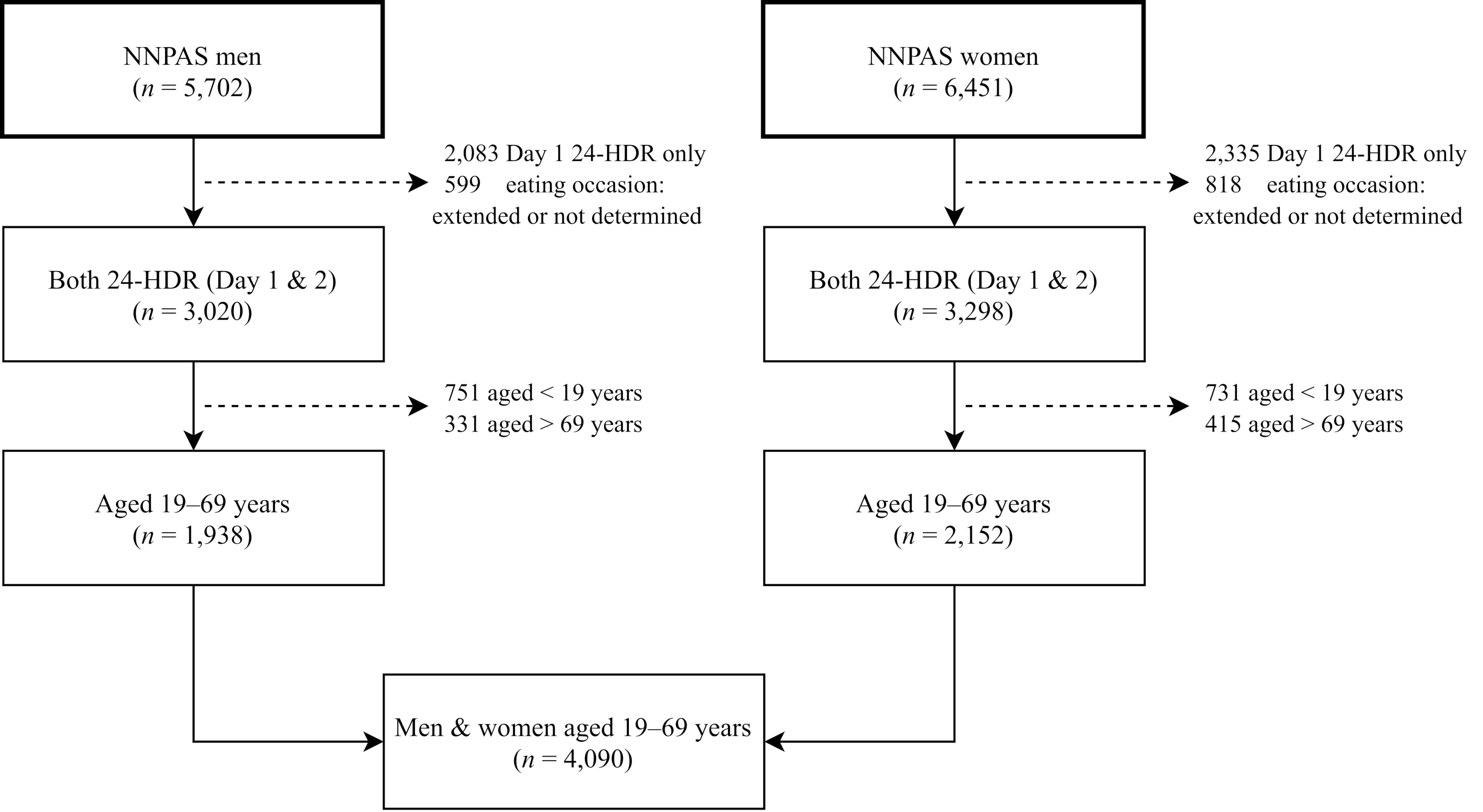


Figure S1. Participant flow chart for NNPAS portion size estimates

We generated portion size information from a pool of 4,090 NNPAS respondents aged 19–69 years (1,938 men and 2,152 women). We included respondents who completed both 24-HDRs (Day 1 and 2). A typical portion size was defined as the usual amount of food consumed on a single eating occasion; therefore, respondents were excluded from portion size estimates if eating occasions were extended over a prolonged period of time or not determined.

*Abbreviations:* NNPAS, National Nutrition and Physical Activity Survey; 24-HDR, 24-hour dietary recall.

Figure S2. Regressions of energy expenditure and estimated energy intake on body weight in males

The DLW database compiled for the 2002 Dietary Reference Intakes consensus report published by IOM served as the criterion for **(A)** the association between measured total energy expenditure and body weight in males (7). Regressions of estimated energy intake on body weight in CDAH were performed on **(B)** the total male sample (females excluded), **(C)** acceptable Goldberg method male energy reporters (±2 standard deviation cut-off, misreporters and females excluded), and **(D)** pTEE method plausible male energy reporters (±1.5 standard deviation cut-off, misreporters and females excluded). In theory, since daily energy intake should equal energy expenditure in weight stable individuals, beta-coefficients and coefficients of determination approximating the criterion imply that estimates are physiologically plausible.

*Abbreviations:* CDAH, Childhood Determinants of Adult Health Study; CI, confidence interval; DLW, doubly labelled water; IOM, Institute of Medicine; pTEE, predicted total energy expenditure.


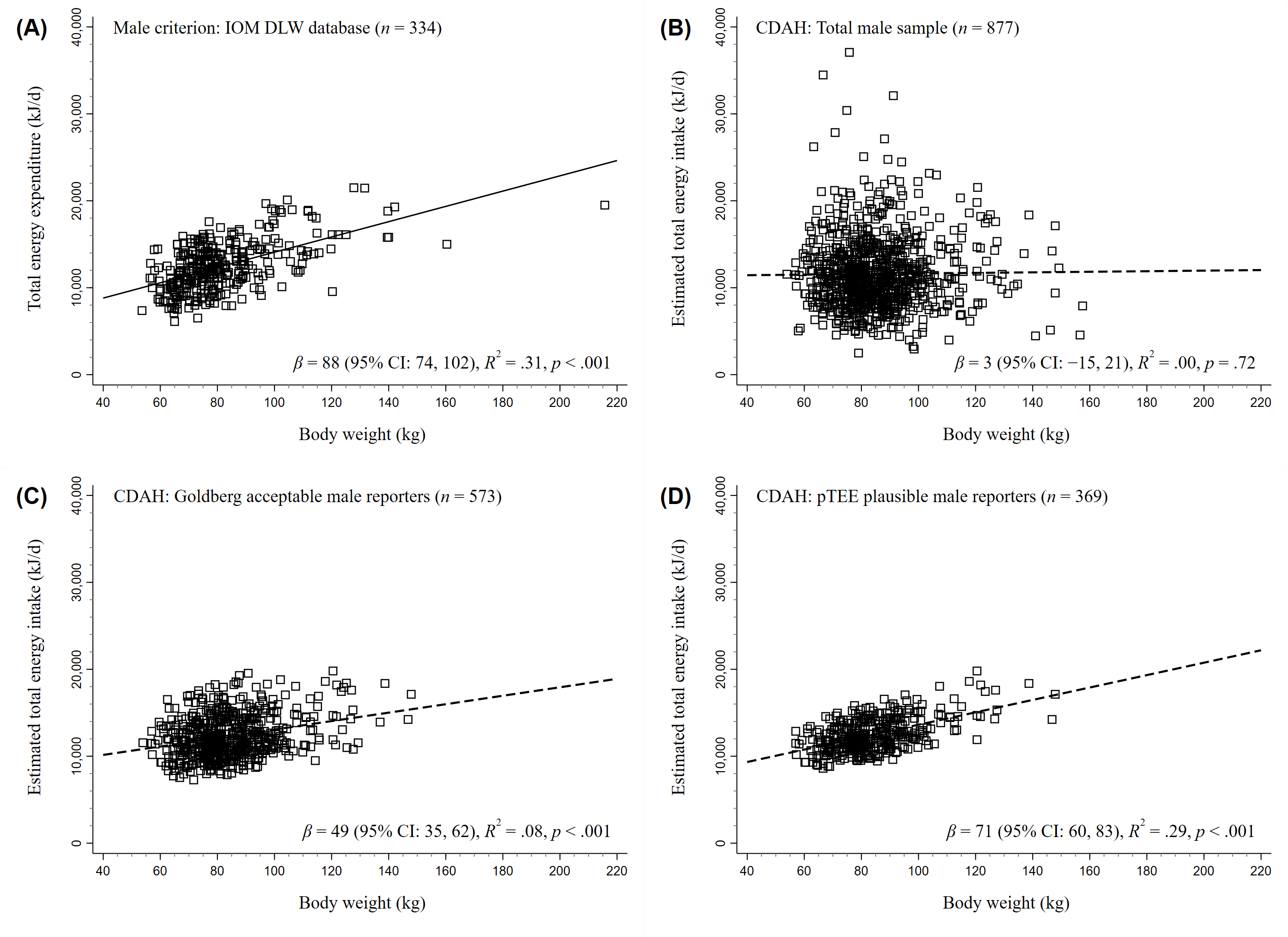


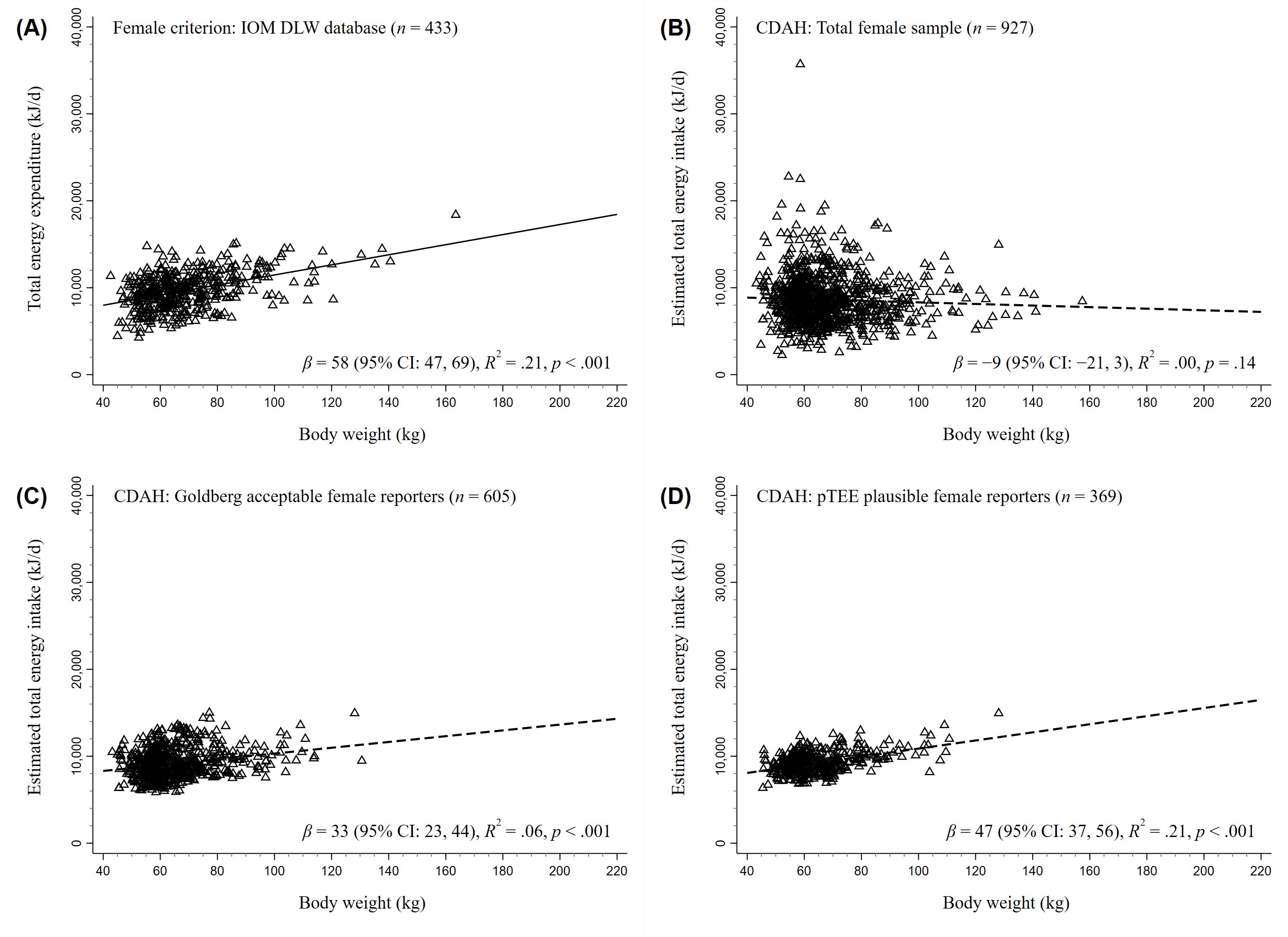


Figure S3. Regressions of energy expenditure and estimated energy intake on body weight in women

The DLW database compiled for the 2002 Dietary Reference Intakes consensus report published by IOM served as the criterion for **(A)** the association between measured total energy expenditure and body weight in females (7). Regressions of estimated energy intake on body weight in CDAH were performed on **(B)** the total female sample (males excluded), **(C)** acceptable Goldberg method female energy reporters (±2 standard deviation cut-off, misreporters and males excluded), and **(D)** pTEE method plausible female energy reporters (±1.5 standard deviation cut-off, misreporters and males excluded). In theory, since daily energy intake should equal energy expenditure in weight stable individuals, beta-coefficients and coefficients of determination approximating the criterion imply that estimates are physiologically plausible.

*Abbreviations:* CDAH, Childhood Determinants of Adult Health Study; CI, confidence interval; DLW, doubly labelled water; IOM, Institute of Medicine; pTEE, predicted total energy expenditure.

# Energy misreporting calculations

Here, we present full calculations detailing the application of the Goldberg and predicted total energy expenditure (pTEE) methods for evaluating energy misreporting. The Goldberg method was proposed in 1991 (1) and revised in 2000 (2), with an accompanying performance paper published on each occasion (3,4). The pTEE method was introduced in 2002 (5) and updated in 2005 (6), and implements the Institute of Medicine’s prediction equations for total energy expenditure (7). We adopt the thermochemical calorie definition when converting kilocalories to kilojoules (1 kcal = 4.184 kJ).

## Goldberg method

The Goldberg method identifies energy misreporters according to their estimated energy intake to basal metabolic rate (EI:BMR) ratio. The Schofield equation conventionally estimates basal metabolic rate (8); however, it may be unsuitable for overweight and obese populations due to overestimation (9). The Mifflin-St. Jeor equation generally reports a higher accuracy rate for estimating basal metabolic rate in obese individuals relative to other equations (10). As such, this paper applies the Mifflin-St. Jeor equation to estimate basal metabolic rate (BMR) (11):

$$\text{Women, BMR (kcal) }\text{= 9.99 × weight} \left( \text{kg} \right)\text{ + 6.25 × height (cm)}-4.92\times age \left( y \right)-161\text{}$$

$$\text{Men, BMR (kcal) = 9.99 × weight} \left( \text{kg} \right)\text{ + 6.25 × height (cm)}-4.92\times age \left( y \right)+5$$

**(Eq. 1)**

The Scientific Advisory Committee on Nutrition determined a population (median) physical activity level of 1.63 by pooling total energy expenditure measurements from two population-based doubly labelled water studies: Beltsville and OPEN (12). Thus, a physical activity level of 1.63 served as a point of reference when assessing group-level energy misreporting and is suggestive of a low activity lifestyle. Individuals considered less active may be allocated a physical activity level of 1.49 (25^th^ centile) broadly equating to a sedentary lifestyle, while more active individuals are allocated a physical activity level of 1.78 (75^th^ centile) indicating a moderate activity lifestyle (12). The EI:BMR ratio and physical activity level are directly comparable in large samples (*n* > 500) (2).

At the individual-level, Childhood Determinants of Adult Health Study participants designated low, moderate and high activity categories, according to the long-form International Physical Activity Questionnaire (IPAQ) scoring protocol (13), were assigned physical activity levels of 1.49, 1.63 and 1.78, respectively. The creation of a 95% confidence limit about each physical activity level (PAL) is by:

$$\text{PAL × exp}\left[ \pm2 SD\times\frac{(S\div100)}{\sqrt{n}} \right]$$

**(Eq. 2)**

, where *n* is the number of study participants, and *S* is a factor that accounts for measurement error and variance in estimated energy intake and basal metabolic rate, and energy requirement. The *n* for individual-level misreporting assessment is 1, therefore removing the √*n* term. The calculation of *S* is by:

$$S=\sqrt{\frac{\mathrm{CV}_{\mathrm{wEI}}^{2}}{d}+\mathrm{CV}_{\mathrm{wB}}^{2}+\mathrm{CV}_{\mathrm{tP}}^{2}}$$

**(Eq. 3)**

, where CV_wEI_ is the within-person coefficient of variation in estimated energy intake, *d* is the number of diet assessment days, CV_wB_ is the coefficient of variation for predicted compared to measured basal metabolic rate, and CV_tP_ is the total coefficient of variation in total energy expenditure (which includes within- and between-person variation and measurement error). Values suggested by Black *et al.* (2) were used in Equation 3 (CV_wEI_ = 23%, CV_wB_ = 8.5%, CV_tP_ = 15%). We considered *d* to be infinity since a food frequency questionnaire reflects habitual intake, and a *d* > 21 has minimal impact on the estimated confidence limit range (2); thus, removing the term for CV_wEI_ (23^2^ / ∞ = 0). Participants with EI:BMR ratios below and above estimated physical activity level confidence limits (for 1.49, 1.63 and 1.78) are considered under- and over-reporters, respectively.

## pTEE method

The pTEE method identifies energy misreporters according to their estimated energy intake to predicted total energy expenditure (EI:TEE) ratio. Total energy expenditure (TEE) was estimated using predictive equations based on sex and body mass index (BMI) published by the Institute of Medicine for adults aged ≥ 19 years (7):

$$Men, BMI<25, TEE=661.8-\left( 9.53\times age \right)+PA\times\left( 15.91\times weight \left[ \mathrm{kg} \right]+539.6\times height \left[ m \right] \right)$$

$$Women, BMI<25, TEE=354.1-\left( 6.91\times age \right)+PA\times\left( 9.36\times weight \left[ \mathrm{kg} \right]+726\times height \left[ m \right] \right)$$

$$Men, BMI\geq25, TEE=1085.6-\left( 10.08\times age \right)+PA\times\left( 13.7\times weight \left[ \mathrm{kg} \right]+416\times height \left[ m \right] \right)$$

$$Women, BMI\geq25, TEE=447.6-\left( 7.95\times age \right)+PA\times(11.4\times weight \left[ \mathrm{kg} \right]+619\times height \left[ m \right])$$

**(Eq. 4)**

, where PA is a physical activity coefficient. Participants are allocated a PA according to their physical activity category: sedentary (≥ 1 to < 1.4), low active (≥ 1.4 to < 1.6), active (≥1.6 to <1.9), and very active (≥ 1.9 to < 2.5) (7). Childhood Determinants of Adult Health Study participants considered to have low, moderate, and high physical activity levels according to IPAQ scoring protocols (13) were assigned to sedentary, low active, and active physical activity level categories, respectively.

The creation of a ±1.5 SD confidence limit to screen EI:TEE ratios is by:

$$\pm1.5 SD=\sqrt{\frac{\mathrm{CV}_{\mathrm{rEI}}^{2}}{d}+\mathrm{CV}_{\mathrm{pER}}^{2}+\mathrm{CV}_{\mathrm{mTEE}}^{2}}$$

**(Eq. 5)**

, where CV_rEI_ is the within-person coefficient of variation for estimated energy intake, *d* is the number of diet assessment days, CV_pER_ is the within-person error in Equation 4 total energy expenditure prediction equations, and CV_mTEE_ is the within-person variation in total energy expenditure and associated measurement error. The 8.2% value for CV_mTEE_ suggested by Huang *et al.* was used (6). As with the Goldberg method, we considered *d* to be infinity, which removes the expression for CV_wEI_. We calculated a mean CV_pER_ of 9.7% by dividing the standard deviation of residuals (i.e., the standard error of fit) (7) for Equation 4 total energy expenditure prediction equations by the mean predicted total energy expenditure across six sex-BMI strata (men and women: < 25, 25–29.9, and ≥ 30 kg/m^2^). A confidence limit of ±19.1% was estimated using a ±1.5 SD cut-off; therefore, a EI:TEE ratio below 0.809 indicates under-reporting while a EI:TEE ratio above 1.191 indicates over-reporting.

# References

1. Goldberg GR, Black AE, Jebb SA, Cole TJ, Murgatroyd PR, Coward WA, Prentice AM. Critical evaluation of energy intake data using fundamental principles of energy physiology: 1. Derivation of cut-off limits to identify under-recording. *Eur J Clin Nutr* (1991) **45**:569–581.

2. Black AE. Critical evaluation of energy intake using the Goldberg cut-off for energy intake:basal metabolic rate: A practical guide to its calculation, use and limitations. *Int J Obes* (2000) **24**:1119–1130.

3. Black AE, Goldberg GR, Jebb SA, Livingstone MBE, Cole TJ, Prentice AM. Critical evaluation of energy intake data using fundamental principles of energy physiology: 2. Evaluating the results of published surveys. *Eur J Clin Nutr* (1991) **45**:583–599.

4. Black AE. The sensitivity and specificity of the Goldberg cut-off for EI:BMR for identifying diet reports of poor validity. *Eur J Clin Nutr* (2000) **54**:395–404. doi:10.1038/sj.ejcn.1600971

5. McCrory MA, Hajduk CL, Roberts SB. Procedures for screening out inaccurate reports of dietary energy intake. *Public Health Nutr* (2002) **5**:873–882. doi:10.1079/PHN2002387

6. Huang TTK, Roberts SB, Howarth NC, McCrory MA. Effect of screening out implausible energy intake reports on relationships between diet and BMI. *Obes Res* (2005) **13**:1205–1217. doi:10.1038/oby.2005.143

7. Institute of Medicine. *Dietary Reference Intakes for Energy, Carbohydrate, Fiber, Fat, Fatty Acids, Cholesterol, Protein, and Amino Acids*. Washington, United States: National Academies Press (2005).

8. Schofield WN. Predicting basal metabolic rate, new standards and review of previous work. *Hum Nutr Clin Nutr* (1985) **39**:5–41.

9. Horgan GW, Stubbs J. Predicting basal metabolic rate in the obese is difficult. *Eur J Clin Nutr* (2003) **57**:335–340. doi:10.1038/sj.ejcn.1601542

10. Frankenfield D, Roth-Yousey L, Compher C. Comparison of predictive equations for resting metabolic rate in healthy nonobese and obese adults: A systematic review. *J Am Diet Assoc* (2005) **105**:775–789. doi:10.1016/j.jada.2005.02.005

11. Mifflin MD, St Jeor ST, Hill LA, Scott BJ, Daugherty SA, Koh YO. A new predictive equation for resting energy expenditure in healthy individuals. *Am J Clin Nutr* (1990) **51**:241–247. doi:10.1093/ajcn/51.2.241

12. Scientific Advisory Committee on Nutrition. *Dietary Reference Values for Energy*. London, England: The Stationery Office (2011).

13. IPAQ Group. Guidelines for data processing and analysis of the International Physical Activity Questionnaire (IPAQ)—short and long forms. (2005) Available at: https://sites.google.com/site/theipaq/ [Accessed April 7, 2020]
